# Metals-triggered compound CDPDP exhibits anti-arthritic behavior by downregulating the inflammatory cytokines, and modulating the oxidative storm in mice models with extensive ADMET, docking and simulation studies

**DOI:** 10.3389/fphar.2022.1053744

**Published:** 2022-11-23

**Authors:** Syed Shams ul Hassan, Syed Qamar Abbas, Ishaq Muhammad, Jia-Jia Wu, Shi-Kai Yan, Fawad Ali, Muhammad Majid, Hui-Zi Jin, Simona Bungau

**Affiliations:** ^1^ Shanghai Key Laboratory for Molecular Engineering of Chiral Drugs, School of Pharmacy, Shanghai Jiao Tong University, Shanghai, China; ^2^ Department of Natural Product Chemistry, School of Pharmacy, Shanghai Jiao Tong University, Shanghai, China; ^3^ Department of Pharmacy, Sarhad University of Science and Technology, Peshawar, Pakistan; ^4^ Department of Pharmacy, Kohat University of Science and Technology, Kohat, Pakistan; ^5^ Faculty of Pharmacy, Hamdard University, Islamabad, Pakistan; ^6^ Department of Pharmacy, Faculty of Medicine and Pharmacy, University of Oradea, Oradea, Romania

**Keywords:** actinobacteria, inflammation, arthritis, anti-oxidant, admet, molecular docking

## Abstract

Triggering through abiotic stress, including chemical triggers like heavy metals, is a new technique for drug discovery. In this research, the effect of heavy metal Nickel on actinobacteria *Streptomyces sp.* SH-1327 to obtain a stress-derived compound was firstly investigated. A new compound cyclo-(D)-Pro-(D)-Phe (CDPDP) was triggered from the actinobacteria strain SH-1327 with the addition of nickel ions 1 mM. The stress compound was further evaluated for its anti-oxidant, analgesic, and anti-inflammatory activity against rheumatoid arthritis through *in-vitro* and *in-vivo* assays in albino mice. A remarkable *in-vitro* anti-oxidant potential of CDPDP was recorded with the IC_50_ value of 30.06 ± 5.11 μg/ml in DPPH, IC_50_ of 18.98 ± 2.91 against NO free radicals, the IC_50_ value of 27.15 ± 3.12 against scavenging ability and IC_50_ value of 28.40 ± 3.14 μg/ml for iron chelation capacity. Downregulation of pro-inflammatory mediators (NO and MDA), suppressed levels of pro-inflammatory cytokines (TNF-α, IL-6, IL-Iβ) and upregulation of expressions of anti-oxidant enzymes (GSH, catalase, and GST) unveiled its anti-inflammatory potential. CDPDP was analyzed in human chondrocyte cell line CHON-001 and the results demonstrated that CDPDP significantly increased cell survival, and inhibited apoptosis of IL-1β treated chondrocytes and IL-1β induced matrix degrading markers. In addition, to evaluate the mitochondrial fitness of CHON-001 cells, CDPDP significantly upregulated pgc1-α, the master regulator of mitochondrial biogenesis, indicating that CDPDP provides protective effects in CHON-001 cells. The absorption, distribution, metabolism, excretion, and toxicity (ADMET) profile of the CDPDP showed that CDPDP is safe in cases of hepatotoxicity, cardiotoxicity, and cytochrome inhibition. Furthermore, docking results showed good binding of CDPDP with IL-6–17.4 kcal/mol, and the simulation studies proved the stability between ligand and protein. Therefore, the findings of the current study prospect CDPDP as a potent anti-oxidant and a plausible anti-arthritic agent with a strong pharmacokinetic and pharmacological profile.

## Graphical Abstract

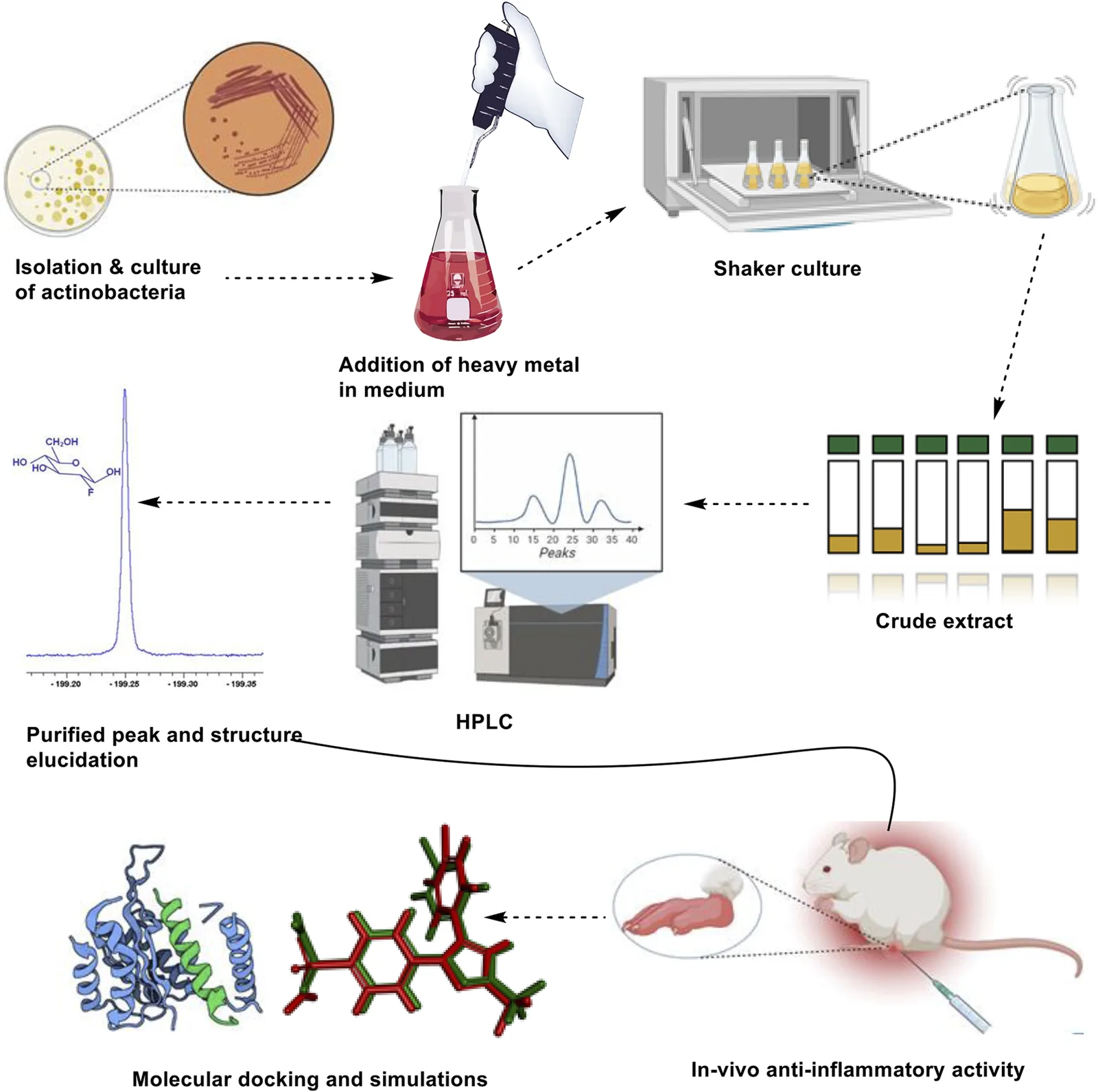

## 1 Introduction

Rheumatoid arthritis (RA) is a chronic inflammatory disease with a global frequency of around 5 per 1,000 persons, according to the World Health Organization. This condition affects women more often than men, and it is most commonly seen in the elderly. It is characterized by the inflammation of various joints and is accompanied by symptoms like periarticular osteopenia, osteitis, and synovitis (Majid et al., 2015; Majid et al., 2018). RA predominantly affects the covering of the synovial joints. It may cause increasing disability, early mortality, significant economic difficulties, and untimely death. The clinical signs of symmetric inflammation include redness, arthralgia, burning, and in severe cases, restriction in range of motion. The most often affected organs by RA are the knees, hands, wrists, and legs. The patients with RA reported feeling soreness and redness in their joints when examined. Most individuals with RA have impairment within 10 years of their diagnosis, making early detection even more critical to limit the inflammatory reaction and subsequent deterioration of joint cartilage and bones (Maryam et al., 2018; Dong et al., 2021).

Prognostic indicators that are associated with the prediction of RA may be divided into two categories: those that are associated with the estimation of signs or indications of RA and those that are associated with the monitoring of bone and cartilage deterioration. It has been shown that the activation of many pathogenic signaling systems occurs in RA, resulting in the progressive and permanent destruction of joint tissue. The growth and durability of fully established RA need the participation of a variety of cells, including dendritic cells, synovial cells, macrophages, and many other immune cells, such as rheumatoid arthritis synovial fibroblasts (RASF), compared to other forms of inflammatory disease (Khan et al., 2021). The various signaling mechanisms involved in the pathogenesis of RA, including Toll-like receptors (TLRs) of the innate immune system, apoptosis-encouraging effector molecules, intracellular kinases, signaling pathways, and proinflammatory cytokine facilitators such as interleukins (IL-6, IL-1β), tumor necrosis factor-alpha (TNF-α), and C-reactive proteins, are all important. Interactions between local cellular components result in the stimulation of cell growth and the degeneration of joints. RASF is not only triggered by primary response genes, proto-oncogenes, as well as intracellular motioning mechanisms that result in the creation and discharge of tissue-destructive chemicals, but proinflammatory cytokines often have a straight relationship with synovial inflammation. The cytokines IL-1β, IL-6, and TNF-α have been elaborated in the expansion of RA through activating the RASF. These cytokines are generated by stimulated macrophages which are richly found in the synovial membrane of the afflicted intersections and are thought to be responsible for the inflammation (Behl et al., 2022). Using nonsteroidal anti-inflammatory medicines (NSAIDs) is advised as first-line therapy to treat arthritis. NSAIDs exert their action by blocking the COX enzyme, which prevents the production of prostaglandins from occurring. Ibuprofen, diclofenac, piroxicam, ketoprofen, naproxen, and flurbiprofen are the most often prescribed NSAIDs to treat chronic pain (Vina et al., 2020). Ibuprofen is provided orally in doses of 200 and 400 mg, depending on the severity of the pain. Traditional synthetic disease-modifying-antirheumatic drugs (DMARDs), as well as biological DMARDs and other newly found treatments, such as targeted synthetic DMARDs, are used in the treatment of RA. During the acute phases, steroids are used to suppress active disease. With fast-developing treatment therapeutic options, a cure for RA has become an aim, with a decrease in pain, joint discomfort, and functional benefits. Despite this, a significant number of patients continue to suffer from serious health impairment, and they often discover that their “own treatment objectives, expectations, and ambitions, as well as those of their clinicians, are seldom satisfied,” according to the American Journal of Medicine. Additional adverse effects of drugs, such as excess weight and insulin resistance, might increase the risk of cardiovascular disease and general morbidity, worsening the problem even more (Vina et al., 2020).

Stressed-out microorganisms have piqued the interest of researchers looking for novel sources of therapeutically useful chemicals (Ul Hassan et al., 2021). While conventional wisdom holds that metals hinder the creation of secondary metabolites, a new study illustrates that metals can excite or increase the activity of powerful medical and nutraceutical important metabolites. Abiotic variables, such as heavy metals, which have been shown to alter the attitude of secondary metabolism in molecular biology and chemical biology, may now transform the synthesis of natural chemicals. DNA sequencing and other innovative procedures have developed secondary metabolites. In pharmaceutical and nutraceutical development, natural ingredients have played a key role. Because of their superior safety and efficacy, natural medications have become more popular over synthetic pharmaceuticals in recent years. Several key challenges inhibit the biotransformation of chemicals into medications, even if microorganisms’ capacity to generate fresh scaffolds looks limitless. Gene clusters and non-activated biosynthetic pathways might be a stumbling hurdle in the identification of secondary metabolites of these crucial medical medicines (Ding et al., 2016; Akhter et al., 2018). The term “sleeping gene clusters” refers to the current status of these gene clusters. This can be explicated through the stimulation of sleeping genes or the creation of compounds possessing stereochemical inclination which facilitates the metal complexation as well as transit in living structures. Metals-triggering plants and microbes are now being used to find new natural products since they are a quick, speedy, and easy way to identify novel chemicals. Some studies are working on expanding the secondary metabolites outline, whereas others are unlocking the microbes intended for their innovative carbon frameworks. The current experiment employed a metal-stress technique to investigate the capacity of *Streptomyces* SH-1327 to yield the production of secondary metabolites with the help of metallic ions. The anti-arthritis activities of the metal-evoked generated molecule was assessed, along with ADMET, docking, and simulations, respectively.

## 2 Materials and methods

### 2.1 Soil sample collection

Marine soil sediment samples were obtained from Zhoushan island, Zhejiang, 100 m from sea shores. The sediments were collected in unpolluted areas near the sea shores on Zhoushan Island. The samples were then taken to the lab and kept at 4°C in the refrigerator.

### 2.2 Isolation and storage of SH1327

To obtain a 10^–1^ dilution, the fresh soil sample of about 1–2 g was immediately subjected to the pre-sterilized glass vials and diluted with simulated seawater. The mixture was sonicated for 1 min to liberate microorganisms tied to the soil particles, then shaken for 15 min at room temperature. The serial dilutions of up to 10^−2^, 10^–3^, and 10^–4^ were then prepared. To prevent fungal contamination, nystatin (0.05 g/L) was added to the pre-prepared isolation media (Gause’s synthetic agar). Following the preparation of the dilutions, 100 μl aliquots of each dilution were inoculated on each media and disseminated with the pre-sterilized spreader. For 15–20 days, the plates were incubated at 28°C. Purified colonies of actinobacteria were collected and kept on agar media and stored at 4°C. The actinobacteria were identified by their morphology, most commonly their colors. Based on the ITS 16S segment, the SH-1312 strain was recognized as a *Streptomyces sp*.

### 2.3 Metal stress and normal cultivation

Metal stress as well as regular colonies of strain SH-1327 were tested in sequence using 500 ml Erlenmeyer flasks containing 200 ml Gause’s media at 28°C in a rotatory shaker at 180 rpm for a total of 10 days in the shaker. In two flasks, a conventional batch growth of variant SH-1327 was performed as a control to ensure that the results were accurate. The stressed culture medium for SH-1327 was supplemented with different concentrations of CuCl_2,_ and NiCl_2_ as 0.5 mM, 1 mM, 2 mM, and 4 mM/L, respectively. To remove the mycelium from the cultural medium, the EtOAc was also added twice having the same concentration.

### 2.4 HPLC technique and refinement of stress metabolite

The HPLC was performed by reversed-phase HPLC-UV (1,290 infinity II HDR-DAD Agilent technologies) using a C_18_ column in order to analyze the separated chemicals. HPLC was set up with 210 wavelengths with H_2_O/MeOH gradients of 20%–100% for 0–30 min and 100% MeOH for 30–50 min, with a constant flow speed of 0.8 ml/min and a steady state frequency of 0.8 ml/min. Next, the stress-induced metabolite was isolated using HPLC at a constant flow rate of 9 ml/min with an 80% MeOH mobile phase.

### 2.5 Extraction and isolation

The 30 L of fermented broth were extracted using EtOAc (2 × 200 ml). Analytical HPLC was used to analyze the crude residue afterward the solvent was completely evaporated and the methanol was added to it and centrifuged for 10 min at 120,000 rpm. Initial screening was done by creating an H_2_O/MeOH gradient of 20%–100% intended for 0–30 min after the HPLC. At 18.13 min, a new peak was discovered. On the Preparative HPLC, the chemical was purified by a steady dissolution medium of 80% MeOH, “MeOH: H_2_O (80:20).” By studying literature, the structure of the strained stressed compound was oriented as a cyclo-(D)-Pro-(D)-Phe (CDPDP) (t_*R*_ = 14 min, 9.2 mg) (Brown and Teller, 1976).

### 2.6 Anti-oxidant assay

To evaluate the antioxidant potential of a stress compound, a multifaceted antioxidant evaluation of a stress compound was performed *via* a DPPH scavenging assay, OH^●^ radical scavenging assay, NO^●^ scavenging assay, and Iron chelating assay by following pre-described protocols (Majid et al., 2018).

### 2.7 *In vitro* anti-inflammatory activity

The anti-inflammatory efficacy was determined by employing the suppression of the albumin denaturation process, which was modified somewhat from the Leelaprakash and Mohan Dass (2011) methodology with minimal modifications. The test consists of reaction mixture of chemicals and a 1% aqueous phase of bovine albumin fraction, with the pH of the reaction mixture adjusted to 7.4 before running the test. After incubation of the sample at 37°C for 20 min, heating to 51°C for 20 min was done. After cooling the samples, turbidity was measured at 660 nm. The experiment was repeated thrice and the % inhibition of protein denaturation was calculated as follows:

$$Percentage\;inhibition=\frac{\left(Abs\;Control\;–Abs\;Sample\right)}{Abs\;Controlo}x\;100$$

### 2.8 Chondrocytes cell line and cell culture

CHON-001 cells were purchased from ATCC (Cat No. CRL-2846) and cultured according to instructions. Cells were grown in a complete DMEM medium enhanced with 1% P/S and 10% FBS and incubated in a 5% CO_2_ incubator at 37°C. Cells were grown up to 80% confluence and each experiment was performed after three passages.

#### 2.8.1 Trypan blue cell exclusion method

Cell viability was ensured with the dye exclusion method. Firstly, 1 × 10^5^ cells from each group were seeded in 24 well cell culture plates and incubated in a 37°C incubator for 24 h and 48 h. Cells were harvested, washed with PBS, and dyed with 0.4% trypan blue for 10 min. Haemocytmeter was used to count the viable (non-stained) and dead (blue-stained) cells. The experiment was performed twice to get the accurate number of viable cells.

#### 2.8.2 Quantitative- real-time PCR

1 × 10^5^ CHON-001 cells/group were grown in 24 well plates until they become 80% confluent. Cells were trypsinized as well as washed with PBS after they were harvested. Further, they were collected and RNA was extracted by using the RNA extraction Trizol method (Invitrogen Trizol reagent cat # 15-596-018). Utilizing a reverse transcription kit, RNA was transformed into cDNA. Syber green PCR Kit (Maxima SYBR green flourescein qPCR master mix (2x) cat #k0243) was used for Q-PCR and results were analyzed using Graph Pad Prism version 7.0. The cycling temperature for PCR is mentioned in table S2. List of primers for quantitative real-time PCR.

##### 2.8.2.1 Target genes cat. No (Qiagen)

The original primer sequence of qPCR is mention in Supplementary Table S1.

*BCL2* QT00000721.

*MMP-1* QT00014581.

*MMP-3* QT00060025.

*Col2a1* QT00049518.

### 2.9 *In vivo* studies

#### 2.9.1 Animals

Adult male albino mice (4–5 weeks old; 24 ± 4 g) were bought from the National Institute of Health (NIH), Islamabad, Pakistan. The animals were kept with good care and with proper food supplements according to the ethical guidelines. All behavioral and biochemical tests were conducted following the guidelines of “The animal care guidelines of QAU” in a sterilized clean environment. The study was carried out after approval from the Bioethical Committee of QAU Islamabad with Ref. No: BCE- DOP-QAU. To avoid any experimental bias, the animals were chosen at random and the experiment was conducted using double blindness. Minimal mice were used for each group in this investigation, and the researchers took great care in minimizing the pain and distress of laboratory animals (Khan et al., 2021).

##### 2.9.1.1 Experimental design

The experimental animals were separated into five groups, each with five mice, and maintained in aluminum cages under regulated conditions (12 h dark/light cycle, 25°C temperature). Prior to usage in experiments, all of the animals were fed a regular diet by following the standard protocols. The grouping was accordingly done as follows in all animal models.

Group-I: Normal (Control).
Group-II: Negative (Control).
Group-III: Positive (Control).
Group-IV: CDPDP (20 mg/kg).
Group-V: CDPDP (40 mg/kg).

#### 2.9.2 Acute inflammatory models

##### 2.9.2.1 Carrageenan-encouraged inflammation impediment in mice

The anti-inflammatory ability of CDPDP was assessed using a rat paw edema model caused by carrageenan (Ahsan et al., 2022). The medications were given 1 hour before being given 1 ml/kg of carrageenan suspension (0.9 percent w/v in saline) in each rat’s left hind paw. Using a plethysmometer, the volume of the paws was measured over time (1, 3, 6, 12, 24 h). Results were calculated as:

$${\text{Edema}\;\text{volume}=\text{PV}}_{t}{-\text{PV}}_{c}$$

Where PVt is paw volume (ml) at a certain time following carrageenan administration, before carrageenan administration, while PVc is paw volume (ml).

$$\text{Percent}\;\text{inhibition}={\text{EV}}_{c}{-\text{EV}}_{t}/{\text{EV}}_{c}\times 100$$

Where EVc is the control group’s edema volume. The edema volume of the treated group is denoted by EVt.

##### 2.9.2.2 Croton oil-induced ear edema constraint

Minor modifications were done to induce edema in the methodology of Reanmongkol et al. (2009). Mice’s right ears were infected with the irritating agent (5% croton oil) when 100 µl of an anesthetic solution was applied to the skin. Acetone was used as a delivery method for the medication. Croton oil was applied to the right ear 1 h before the application of CDPDP (5.0 mg/ear each). Anti-inflammatory medication ibuprofen (1 mg/ear) was used as a control. After the mice were sacrificed 4 h later, a plug (7 mm diameter) was taken from both the treated and untreated ears. The difference in weight in the two plugs was used to compute the percent inhibition of edema.

##### 2.9.2.3 Croton oil-induced anus edema inhibition

Croton oil-induced edema of the anus was accomplished by adjusting Reanmongkol et al. (2009) approach. To induce anal necrosis, an anus-inducing agent was applied to the anus of mice for 10 s (0.2 ml of croton oil 6% in diethyl ether). Besides, CDPDD (40 and 20 mg/kg) and Ibuprofen (10 mg/kg) were administered orally OD for 3 days after directing croton oil. On the fourth day, the diameter within each heavily sedated mice anus (in millimeters) was measured with a vernier caliper, and the results were recorded.

#### 2.9.3 Chronic anti-inflammatory model

##### 2.9.3.1 CFA-induced inflammatory pain model

To investigate the properties of extracted CDPDP in the chronic inflammatory model. According to the study design, the experimental mice were randomly selected into five groups and the CFA-induced chronic pain model was employed in mice, as described above. A complete Freund’s adjuvant (CFA) provoked arthritic model (Negative control) was created by injecting 25 μl of CFA into the mouse paw intraplantarly (i.pl). When arthritis had fully developed, the CDPDP’s anti-inflammatory activity was evaluated using a therapeutic scoring rate and an observational study. Ibuprofen was given to the positive control group, whereas CDPDP (20 mg/kg and 40 mg/kg) was given to the treatment groups. The effects of CDPDP on the chronic model were examined for 22 days, with the clinical score being tracked from 14 to 22 days by measuring the swelling of the paws before edema induction on day one and subsequently (Khan et al., 2022).

##### 2.9.3.2 Paw edema parameter in mice

The chronic (CFA-induced inflammation) model was used for the assessment of the pharmacological effects of a CDPDP (20 mg/kg) and CDPDP (40 mg/kg) compared to ibuprofen. The Edema parameter was measured using a dial thickness gauge after inflammation was induced in the mouse paw (Mitutoyo Corporation, Japan).

##### 2.9.3.3 Thermal hyperalgesia

On a heat plate, a CDPDP (20 mg/kg) with different doses was evaluated for thermal hyperalgesia. Mice were placed on a heat plate with a temperature of 37 ± 0.5°C to explore thermal hyperalgesia. Responses were obtained at 0,7,14, 16, 18, and 20 days after treatment in the CFA -induced inflammatory model. However, the tickling, grimacing, and sucking of the paw over the heat plate were all positive responses for thermal hyperalgesia, and the total time the paw lasted on the heating plate was also measured. The investigational group animals were divided into five groups, as previously mentioned. A CDPDP and a commercially available Ibuprofen were used to compare the effects against chronic thermal hyperalgesia (Eo et al., 2014).

##### 2.9.3.4 Mechanical allodynia

Mechanical allodynia was tested in the experimental animals using the Von Frey Filament technique to assess pain sensibility. All the experimental animals were placed in a plastic box with a small mesh floor to check the dorsal surface of the right hind paw for mechanical allodynia. The Von Frey Filament method was used to explore mechanical allodynia in a double-blinded study. The mice were separated into five groups, and the protocol was followed just as before. Mice withdrawal reflex was observed (three times out of five) through the filament to investigate the pain of the right hind paw as a positive reaction. The suppressive impact of the CDPDP (20 mg/kg) was further tested against CFA-induced paw edema in mice (Yu et al., 2012).

##### 2.9.3.5 Collection of blood and organ sampling

Anesthetic agents (ketamine and xylazine at 16 mg and 60 mg, respectively, intraperitoneally) were used to anesthetize all of the experimental animals before the samples were taken after the activity. Immediately after the heart puncture, a blood sample was obtained and centrifuged for 5 min at 4050 RCF at 4°C. To perform several biochemical tests, the clear serum obtained from the blood sample was employed. A formalin solution of 10% was added to the Eppendorf tubes that contained the tissues and organs for histological and radiological examination (Ullah et al., 2018).

##### 2.9.3.6 Hematology

The blood studies including the count of red blood cells (RBCs), white blood cells (WBCs), as well as platelets, were done using a Neubauer hemocytometer (Feinoptik, Germany). The amount of hemoglobin (Hb) in the blood was determined with the help of Sahli’s hemoglobin meter. The modified Westergren method was used to determine the rate of erythrocyte sedimentation (ESR) (Nair et al., 2012).

### 2.10 Biochemical assays

#### 2.10.1.Nitric oxide determination in plasma

Nitric oxide (NO), which is recognized as a basic characteristic of inflammation, is used to determine the degree of inflammation. It is a key sign of the ongoing inflammatory process and can be linked to the severity of the inflammation (Kim et al., 2002). NO levels in mice plasma were examined in this investigation to see if CDPDP had an impact on NO generation. All the experimental procedures were identical to those described in the preceding sections. To achieve a homogenous mixture, plasma (25 μl), an equal volume of normal saline, and Griess reagent were combined. The mixture was refrigerated for 1 h before the NO concentration was determined using a 450 nm microplate reader (Batool et al., 2017).

#### 2.10.2 Effect of CDPDP on the antioxidant system

The liver is the most sensitive region to oxidative stress, and it expresses a variety of antioxidant systems to combat it, including glutathione (GSH), glutathione S transferase (GST), and catalase (Hitchon and El-Gabalawy, 2004). The effect of CDPDP on antioxidant enzymes such as catalase, GST, and GSH was studied. The effect of commercially available ibuprofen and a specially extracted compound on these pathways was investigated.

##### 2.10.2.1 Reduced GSH assay

GSH levels were determined using paw tissue homogenates. Paw tissues were put in 2–3 ml of phosphate buffer and homogenized for 10–15 min at 13,500 rpm. The homogenate was centrifuged upto10 min at 9,080 rcf, the sediment was discarded and the supernatant was collected and preserved for further analysis. Following that, the Ellman reagent was used to calculate GSH (Khan et al., 2021). The results were represented as a percentage of the total GSH released.

##### 2.10.2.2 GST assay

The purpose of this test was to see if the GST enzyme could conjugate GSH with 1-chloro-2-4-dinitrobenzene. Their activity was measured using a microplate reader at 340 nm. An assay combination without supernatant was used as a blank to check for nonspecific binding (Batool et al., 2017).

##### 2.10.2.3 Catalase assay

The ability of catalase to degrade hydrogen peroxide was measured using a progressive decrease in absorbance at 240 nm. The percentage of catalase produced per gram of tissue was calculated (Batool et al., 2017).

##### 2.10.2.4 Lipid peroxidation assay

Malonaldehyde (MDA) is an important element to study in lipid peroxidation (LPO) assays, however, the NSAIDs and CDPDP are thought to impact fat accumulation and enhance the effect on reactive oxygen species (ROS). The LPO was also tested using a CDPDP-based extract and commercially available ibuprofen. The percentage of MDA generated per gram of tissue was calculated (Kim et al., 2002).

##### 2.10.2.5 LFT’s and RFT’s

With standard AMP diagnostic kits, the serum and urine of experimental mice were tested for several markers including Urea, Creatinine, AST, ALT, ALP, bilirubin, and albumin using a variety of different tests (Stattogger Strasse 31b 8,045 Graz, Austria). The Bradford method was used to screen the protein concentration (Nair et al., 2012).

#### 2.10.3 Measurement of cytokines

Assays were performed using an ELISA kit (BD Bioscience Inc. San Diego, CA ELISA kit) to measure cytokine concentrations. Tissue samples were taken from all experimental groups under the instructions (Cui et al., 2020). The right hind paw was amputated on the last day of activity, and tissue proteins were isolated with a Phosphate buffer solution consisting of 0.005% tween 80, 0.4 M sodium chloride, and a protease inhibitor at a concentration of 100 mg/ml of solution. The samples were homogenized uniformly before being centrifuged for 5 min at 5,000 rpm to collect the supernatant, which was then kept at −80°C for further analysis.

### 2.11 ADMET analysis

ADMET (Absorption, distribution, metabolism, excretion, and Toxicity) are the essential measurement tools for any compound before being elected as a drug candidate. The online web tool swiss ADME (http://www.swissadme.ch/index.php) was used to obtain ADMET properties of the stress compound (Daina et al., 2017), and the Online web tool pkCSM (http://biosig.unimelb.edu.au/pkcsm/prediction) was used to predict the pharmacokinetic scores (Zhuo et al., 2020).

#### 2.11.1 Prediction of cardiac toxicity

Blockage of the hERG K^+^ channels has been linked to fatal cardiac arrhythmias. To predict cardiac toxicity, a free accessible online service pred-hERG 4.2 (http://predherg.labmol.com.br) was used for its early detection of potential hERG blockers and non-blockers (Braga et al., 2015).

#### 2.11.2 Skin sensitization

Skin sensitization is a crucial phase in allergic contact dermatitis, a complex immune-mediated inflammatory skin reaction. Till now there are no *In-vitro* techniques to evaluate the capacity of any compound for its skin sensitisation So, we have conducted the skin sensitization evaluation of a CDPDP through an *in-silico* technique from online web portal http://predskin.labmol.com.br/(Braga et al., 2017).

### 2.12 Molecular docking

#### 2.12.1 Protein preparations

The 3 D crystal structure of Interleukin-6 protein (PDB ID: 1il6) and Tumor Necrosis Factor-alpha (PDB ID: 1tnf) was retrieved from the RCSB Protein data bank (https://www.rcsb.org/). The resulting structure was refined by utilizing the Protonate3D program (Dallakyan and Olson, 2015) to add partial charges and energy minimization by using the Molecular Operating Environment to pick the MMFF94x force-field (MOE, Version 2018.01). The MOE software’s site finder function was used to locate the active cavity in the target protein.

#### 2.12.2 Ligand preparation

CDPDP was selected to screen their potential inhibitory effect against Interleukin-6 protein (PDB ID: 1il6) and Tumor Necrosis Factor-alpha (PDB ID: 1tnf) protein using *in silico* tools. The ligands were optimized *via* Protonate3D and energy was minimized before the addition of the MOE ligand database. The 2 D chemical structure of the selected ligands was drawn using ChemDraw. The standard ibuprofen was used as a reference to compare the inhibitory effect of the selected plant-based compounds.

#### 2.12.3 Docking analysis

CDPDP was docked with interacting residues of PDB ID: 1il6 and Tumor Necrosis Factor-alpha (PDB ID: 1tnf) protein by using the docking algorithm of MOE software. The MOE program used the “Triangular Matcher” technique to build a minimum energy structure, which was then used as the default ligand insertion strategy. Rescoring of simulated poses was done using the London dG scoring tool. Based on their root mean square deviation (RMSD) and S-score binding affinity values, CDPDP with the best and top conformation was identified after docking. The MOE LigX tool was used to examine the 2D plots of ligand-receptor interactions, and the best-docked complexes were displayed. MOE was used to generate 3D images of protein inhibitor complexes.

### 2.13 Molecular docking simulations

The MDS analysis was used to determine the ligand-protein stability interaction. The structure of the macromolecules transitions to the functional importance of the complex was also studied through MD simulations. To estimate the binding of the ligand in the cellular environment, simulations gather valuable atom movement concerning time using Newton’s standard motion equation. The MD simulations of the CDPDP-IL-6 and Ibuprofen-IL-6 were performed for 100 ns using the Schrodinger suite’s Desmond v:3.6 New York, United States module (Shams ul Hassan et al., 2022). The preliminary structure of MD simulations was followed by an established procedure where protein atoms were 10 Å away from the box, and the interaction of the complex derived from MD was the initial structure of respective MD simulations. Wizard of Maestro was used to minimize and optimize the ligand-receptor complex. Using the System Builder tool, a solvent standard TIP3P with an orthorhombic box was chosen for the systems. For simulation analysis, the OPLS 2005 was employed. The model’s physiological circumstances were modified by the addition of 0.15 M NaCl (Ferreira et al., 2015). Finally, for all MDSs, simulations were run at a constant temperature of 300 K and pressure of 1 atm with an NPT ensemble. Furthermore, every 100 ps interval, the MDS trajectories were recorded. To measure the stabilities of protein-ligand interactions in simulations, the values of root mean square deviation (RMSD), RMSF, the radius of gyration, and SASA were determined for both protein and ligand. MDSs were run three times for each complex, with the same parameters each time.

#### 2.13.1 MMGBSA calculations

The prime tool in maestro was used to reduce the ligand-receptor complexes. After minimization, the binding free energies (∆*G*) of complexes were calculated using molecular mechanics generalized Born surface area (MMGBSA) before (0 ns) and after (100 ns) simulation. An OPLS_2005 force field was used throughout the computation of binding free energy.

### 2.14 Statistical analysis

The values for each test were expressed as the mean +standard deviation. Using Statistix 8.1 and Tukey’s multiple comparison tests based on parametric examination of variance, the magnitudes of altered treatments to mice *in vivo* were evaluated. *p* ≤ 0.05 was used to determine statistical significance for activity.

## 3 Results

### 3.1 HPLC profile of SH1327 with metal treated and untreated extracts

It was observed that an actinobacteria-stressed metabolite could be extracted using an HPLC configuration that took into account elution modes, mobile phase, monitoring wavelength, and column temperature. Earlier studies show that metals such as Nickel (Ni^2+^), zinc (Zn^2+^), Copper (Cu^2+^), and mixed metals (Co^2+^+Zn^2+^) may induce the production of secondary metabolites in microorganisms. Two distinct types of metals nickel and copper were selected as initial elicitors. At 28°C, the culture experiment with strain SH1327 was conducted in Gause’s medium containing ions of nickel and copper with four different starting concentrations. The experiment was carried out at 180 rpm in a rotatory shaker over 18 days. The metal CuCl_2_ did not stimulate any compound in any concretion, however, two novel peaks were triggered in NiCl_2_. Using a blank medium, one medium without strain, one medium without metal, and four sets of medium with different ionic concentrations. We confirmed the changes in the metabolic parameters that occurred when metals were exposed (0.5 mM–4 mM). An EtOAc extraction was performed on the culture broth (2 × 200 ml) by using a gauze filter to separate the mycelium from the culture broth. The chromatogram from HPLC revealed that *Streptomyces* actinobacteria have a unique stress-induced metabolite cyclo-(D)-Pro-(D)-Phe (CDPDP) was identified in metal-treated cultures, while it was not discovered in untreated cultures. In non-metal cultures, it was practically invisible. It was discovered that the blank variant SH-1327 did not create any metabolites when grown under standard conditions.

### 3.2 Identification of metal-elicited metabolite

**FIGURE 1:**
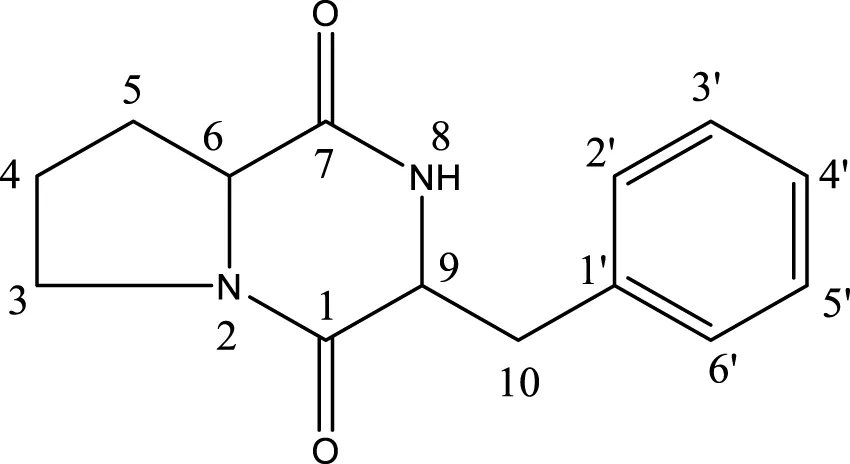
Chemical structure of CDPDP.

After the HPLC chromatogram at 18.13 min, a new peak was discovered (Supplementary Figure S1). On the Preparative HPLC, the chemical was purified by a steady dissolution medium of 80% MeOH, “MeOH: H_2_O (80:20).” By studying literature, the structure of the strained stressed compound was oriented as a cyclo-(D)-Pro-(D)-Phe (CDPDP) (t_*R*_ = 14 min, 9.2 mg) (González-Menéndez et al., 2016) (Figure 1). As of this writing, this is the first case in which metal-elicitation-induced CDPDP was seen.

### 3.3 Antioxidant assay

**FIGURE 2:**
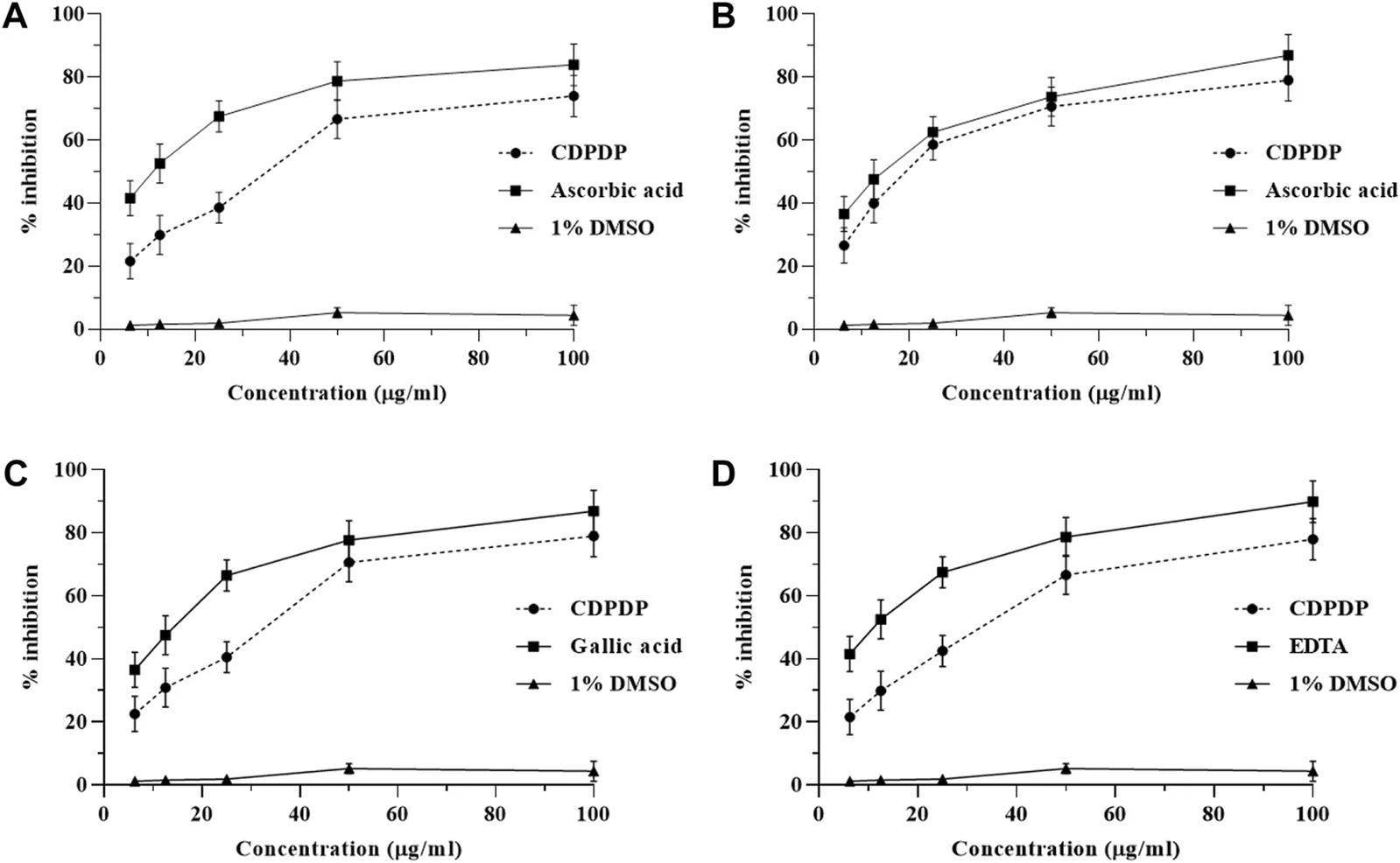
Representation of CDPDP antioxidant activity *in vitro*. Note: **(A)** DPPH radical scavenging properties, **(B)** NO^●^ scavenging activity, **(C)** OH^●^ scavenging activity, and **(D)** iron-chelating % inhibition. All values are represented as Mean ± SD (*n* = 3).

In order to evaluate the potential of CDPDP as an antioxidant agent, multimode antioxidant tests were performed. In the DPPH test, CDPDP displayed promising outcomes, inhibiting the free radicals with 73.99% ± 5.12% at 100 μg/ml ratio, compared to the conventional ascorbic acid (83.81% ± 4.9%). The IC_50_ for CDPDP in DPPH was documented as 30.06 ± 5.11 μg/ml, even though IC_50_ for ascorbic acid was 10.08 ± 3.77 μg/ml. CDPDP inhibited NO free radicals by 76.79% ± 3.98%, compared to 86.89% ± 3.69% for ascorbic acid, with an IC_50_ of 18.98 ± 2.91 for CDPDP and 13.21 ± 3.41 μg/ml for ascorbic acid. Similarly, CDPDP demonstrated significant scavenging activity against OH^●^ radicals, suppressing 78.69% ± 4.93% radicals at a concentration of 100 μg/ml, compared to gallic acid (86.15% ± 3.67%) as a control. CDPDP and gallic acid have been verified to have IC_50_ values of 27.15 ± 3.12 and 12.42 ± 2.72 μg/ml, respectively. The iron chelation ability of CDPDP and standard were examined to assess the chelation strategy of compounds to hunt free radicals. CDPDP seemed to chelate 74.96% ± 5.79% of iron radicals (IC_50_ 28.40 ± 3.14 μg/ml), compared to EDTA with 88.52% ± 4.67% chelation capability and IC_50_ of 10.20 ± 3.16 μg/ml. Figure 2 depicts the entire set of results.

### 3.4 *In vitro* anti-inflammatory potential of CDPDP

The suppression of heat-induced albumin denaturation test was used to assess CDPDP’s anti-inflammatory efficacy *in vitro*. At a concentration of 400 μg/ml, CDPDP inhibited protein denaturation by 68.54% ± 2.97% compared to the standard, ibuprofen (71.48% ± 4.71%). Table 1 presents a summary of the findings.

**TABLE 1:** *In vitro* anti-inflammatory assessment of CDPDP.

Samples	400 μg/ml	200 μg/ml	100 μg/ml
Control	–	–	–
Ibuprofen	71.35 ± 3.89^a^	63.66 ± 3.12^c^	61.53 ± 4.84^cd^
CDPDP	68.54 ± 2.97^b^	57.72 ± 3.54^d^	54.19 ± 2.32^e^

The data values displayed are the mean standard deviation ± (*n* = 3). The means in the column with distinct superscript (a-e) letters are substantially different ((*p* < 0.05).

### 3.5 Effect on carrageenan induced inflammation in mice

The anti-inflammatory capacity of the CDPDP was investigated through the Carrageenan-induced paw edema model (inflammatory model). The results declared that CDPDP has more significant anti-inflammatory activity than the control model. CDPDP (40 mg/kg) displayed determined inhibition of inflammation (78.37% ± 4.92%) at sixth h while CDPDP (20 mg/kg) displayed inhibition with a value of (67.31% ± 5.11%) compared to Ibuprofen (81.27% ± 6.89%). Table 2 presents a summary of the findings.

**TABLE 2:** Effect of CDPDP on carrageenan-induced paw edema in mice.

Groups	Percent edema inhibition				
	1st h	3rd h	6th h	12th h	24th h
Control (Saline)	–	–	–	–	–
Carrageenan control	–	–	–	–	–
Ibuprofen	39.92 ± 2.99^a^	58.87 ± 4.49^a^	81.27 ± 6.89^a^	77.86 ± 6.24^a^	73.36 ± 5.99^a^
CDPDP (20 mg/kg)	18.87 ± 1.69^c^	34.98 ± 2.92^c^	67.31 ± 5.11%^c^	65.78 ± 5.86^c^	58.89 ± 3.68^c^
CDPDP (40 mg/kg)	28.98 ± 2.96^b^	42.82 ± 3.68^b^	78.37 ± 4.92^b^	74.72 ± 6.02^b^	70.29 ± 4.57^b^

The data values displayed are the mean standard deviation ± (*n* = 6). The means in the column with distinct superscript (a-c) letters are substantially different (*p* < 0.05).

### 3.6 Croton oil-induced inflammation inhibition

The anti-inflammatory efficacy of the CDPDP was investigated further using a croton oil-induced ear edema and anal edema model (inflammatory model)*.* The results showed that CDPDP (40 mg/kg) has significant anti-inflammatory actions linked to the control model. CDPDP (40 mg/kg) exhibited determined inhibition of the ear inflammation (71.98% ± 7.78%) while the CDPDP (20 mg/kg) shows inhibition with the score of (61.99% ± 4.07%), compared to Ibuprofen (80.47% ± 4.98%) (Table 3). Moreover, the CDPDP (40 mg/kg) showed maximal inhibition of anal inflammation (69.78% ± 4.88%), while the CDPDP (20 mg/kg) shows inhibition with a score of (62.74% ± 4.06%), compared to Ibuprofen (81.99% ± 6.19%) (Table 3).

**TABLE 3:** Effect of *CDPDP* on croton oil-induced ear edema in mice.

Groups	Weight of left ear (mg)	Weight of right ear (mg)	Edema (∆ mg)	% Reduction of inflammation
a)				
Control (Saline)	80.04 ± 1.4	80.0 ± 2.02	–	–
Croton oil	91.4 ± 1.10	97.50 ± 2.1	6.10 ± 0.74	–
Ibuprofen	86.7 ± 1.67	87.86 ± 2.01	1.16 ± 0.10	80.47 ± 4.98^a^
CDPDP (20 mg/kg)	90.44 ± 1.7	92.73 ± 1.5	2.29 ± 0.20	61.99 ± 4.07^c^
CDPDP (40 mg/kg)	84.34 ± 2.1	86.07 ± 1.8	1.73 ± 0.15	71.98 ± 7.78^b^
b) Effect of CDPDP on croton oil-induced anal edema in mice				
Groups		Anus size (mm)	Edema (mm)	% reduction of inflammation
Control (Saline)		33.82 ± 0.89	–	–
Croton oil		45.92 ± 1.19	12.46 ± 0.7	–
Ibuprofen		35.95 ± 1.02	2.09 ± 0.11	81.99 ± 6.19^a^
CDPDP (20 mg/kg)		39.02 ± 1.59	4.48 ± 0.20	62.74 ± 4.06^c^
CDPDP (40 mg/kg)		38.12 ± 1.38	3.49 ± 0.27	69.78 ± 4.88^b^

The data values displayed are the mean standard deviation ± (*n* = 6). The means in the column with distinct superscript (a-c) letters are substantially different (*p* < 0.05).

### 3.7 Effect of CDPDP on paw edema in CFA-induced chronic arthritis model in mice

**FIGURE 3:**
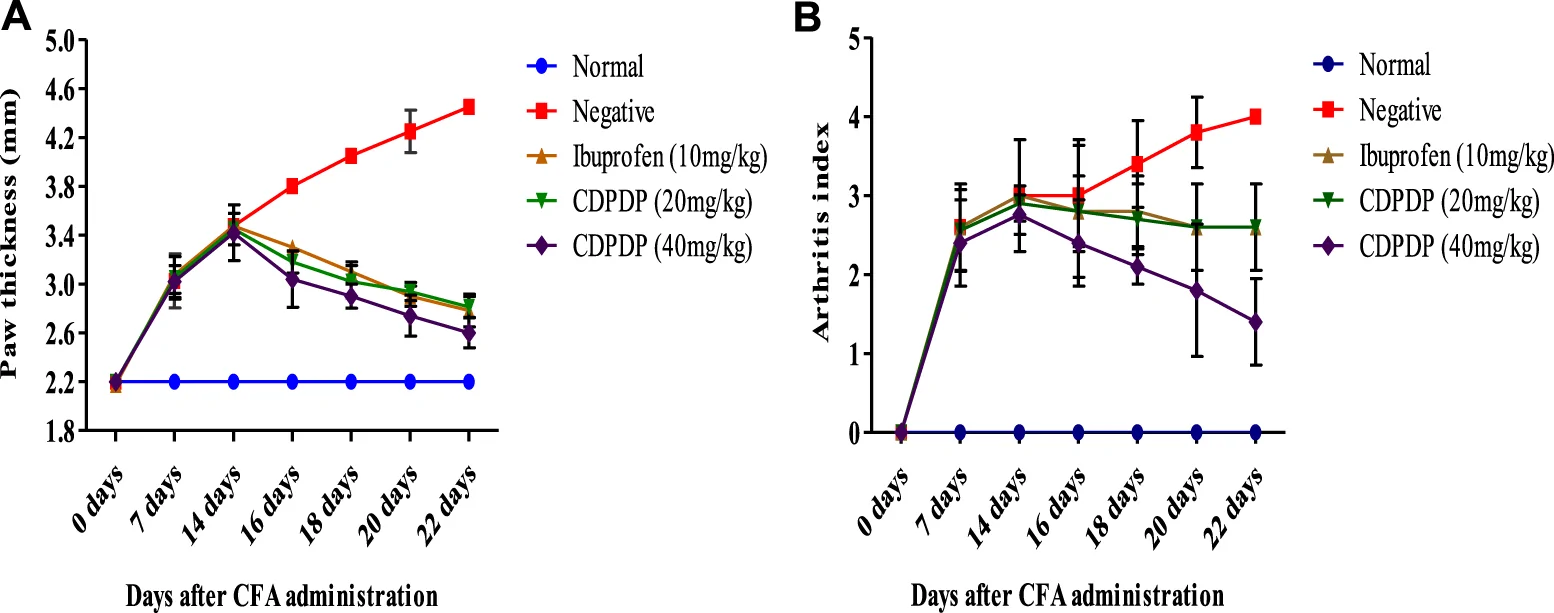
**(A)** Effect of chronic pre-treatment with CPPDP (20 and 40 mg/kg) on paw edema. After CFA injection, paw thickness was measured for a period of 0 up to 22 days. **(B)** Arthritic index where arthritic control (Negative) group has higher arthritic score compared to normal and test groups. Between days 14 and 22, the arthritic index of the animals treated with ibuprofen as well as CDPDP (20–40 mg/kg) exhibited a statistically significant decrease in the treated animals. Data sets are displayed as mean ± SD (*n* = 5) with significance difference at *p* < 0.05.

CFA sub planter injection in the right hind paw of mice resulted in a considerable chronic inflammation that lasted for 22 days. CDPDP (20 and 40 mg/kg) significantly reduced paw edema between days 14 and 22 (Figure 3A). Ibuprofen, a positive control, inhibited paw thickness significantly throughout the arthritis experiment. Arthritic scoring (Figure 3B) is indicating significant anti-arthritic activity of the compound at low and high doses.

### 3.8 Effect of CDPDP pre-treatment on mechanical hyperalgesia and mechanical allodynia

**FIGURE 4:**
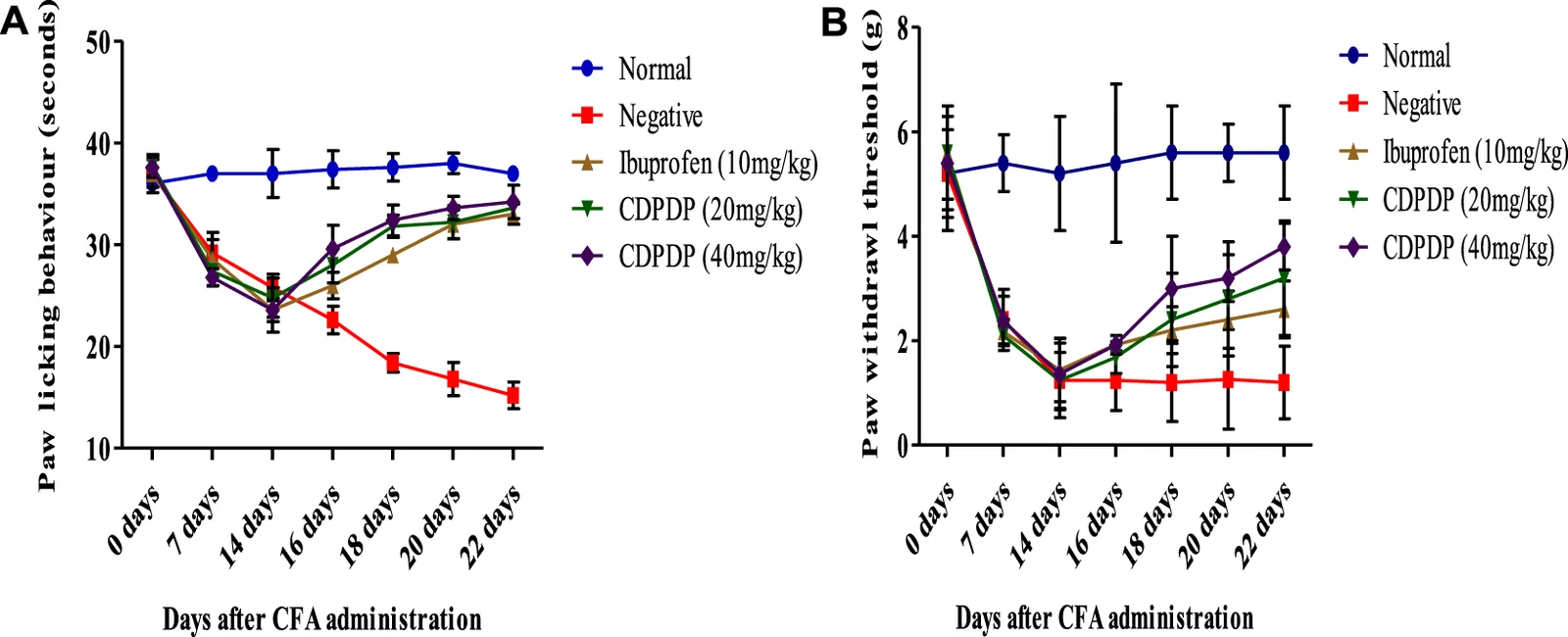
**(A)** Effect of chronic CDPDP pre-treatment on thermal hyperalgesia. The paw licking response was measured post-CFA injection from 0 to 22 days. **(B)** Effect of chronic CDPDP pre-treatment on cold allodynia. The paw withdrawal effect was measured on days 1 To 22 days after CFA administration. The data are expressed as the mean ± SD at *p* < 0.05 and *p* < 0.01.

CDPDP (20 and 40 mg/kg) considerably reduced the mechanical hyperalgesia (*p* < 0.0001) at days 14–22 (Figure 4A). Mechanical nociception was likewise significantly reduced in the CFA-induced group on day 1 and then gradually reduced until day 21 (Figure 4A). Similarly, from day 14–22 of CFA injection, i.pl. treatment of CDPDP (20 and 40 mg/kg) dramatically reduced mechanical allodynia in mice, as seen in Figure 4B. Ibuprofen (10 mg/kg) also reduced CFA-induced mechanical allodynia. At days 14–21, CDPDP (40 mg/kg) revealed the most substantial increase in paw withdrawal threshold when compared to the negative control (Figure 4B).

### 3.9 Effect on arthritis-induced hematological variations

Table 4 shows the outcomes of the arthritis-related hematological changes. The findings revealed that as arthritis progresses, hematological values like hemoglobin (Hb), RBCs, and platelets decrease, while ESR and WBC levels increase in arthritic animals. As indicated in Table 5, CDPDP administration dramatically improved these hematological parameters.

**TABLE 4:** The hematological examination of normal control besides arthritic rats.

Groups	RBCs (×10^6^)/µl	WBCs (×10^3^)/µl	Platelets (×10^3^)/µl	Hb (g/dl)	ESR (mm/h)
Control	6.72 ± 0.067^a^	3.49 ± 0.039^a^	589.79 ± 4.56^a^	10.29 ± 0.8^a^	4.32 ± 0.041^a^
Negative Control	4.09 ± 0.026^e^	6.99 ± 0.030^d^	351.06 ± 2.49^d^	6.29 ± 0.11^d^	9.38 ± 0.058^c^
Ibuprofen	5.46 ± 0.028^d^	6.28 ± 0.019^c^	489.4 ± 02.30^c^	7.77 ± 0.68^c^	4.99 ± 0.016^b^
CDPDP (20 mg/kg)	5.76 ± 0.018^c^	3.76 ± 0.008^b^	550 ± 02.801^b^	8.10 ± 0.46^b^	4.89 ± 0.048^b^
CDPDP (40 mg/kg)	5.99 ± 0.011^b^	3.68 ± 0.012^ab^	568 ± 02.703^ab^	8.18 ± 0.66^b^	4.81 ± 0.068^b^

The data values displayed are the mean standard deviation ± (*n* = 6). The means in the column with distinct superscript (a-e) letters are substantially different (*p* < 0.05).

**TABLE 5:** Effect of CDPDP on liver function tests (LFT’s).

Groups	ALT (U/L)	AST (U/L)	ALP (U/L)	Bilirubin (mg/ml)	Albumin (mg/dl)
a)					
Control	38.93 ± 0.48^a^	25.9 ± 0.34^a^	114.8 ± 1.9^a^	6.79 ± 0.21^a^	6.37 ± 0.24^a^
Negative Control	97.29 ± 3.41^d^	106.1 ± 2.1^d^	346.09 ± 7.3^d^	11.4 ± 0.19^d^	2.69 ± 0.12^c^
Ibuprofen	43.21 ± 2.43^b^	34.0 ± 2.1^b^	121.2 ± 3.7^b^	7.12 ± 0.66^b^	5.97 ± 0.21^ab^
CDPDP (20 mg/kg)	46.74 ± 3.83^c^	37.1 ± 2.4^c^	137.3 ± 4.2^c^	7.59 ± 0.06^c^	5.68 ± 0.13^b^
CDPDP (40 mg/kg)	43.6 ± 0.46^b^	30.9 ± 0.7^b^	120.8 ± 2.7^b^	7.11 ± 0.29^b^	5.99 ± 0.39^ab^
b) Effect of CDPDP on renal function tests (RFT’s)					
Groups	Urea (mg/dl)	BUN (mg/dl)	Creatinine (mg/dl)	GFR (ml/min)	Protein (mg/dl)
Control	51.4 ± 1.87^a^	20.5 ± 0.33^a^	1.48 ± 0.09^a^	0.43 ± 0.03^a^	5.12 ± 0.13^a^
Negative Control	76.1 ± 2.41^d^	50.8 ± 1.9^d^	3.01 ± 0.16^d^	0.07 ± 0.02^e^	1.29 ± 0.11^b^
Ibuprofen	54.9 ± 2.8^b^	26.1 ± 2.4^b^	1.63 ± 0.11^b^	0.36 ± 0.04^ab^	5.09 ± 0.07^a^
CDPDP (20 mg/kg)	57.8 ± 3.5^c^	26.9 ± 2.4^c^	1.65 ± 0.10^c^	0.34 ± 0.03^b^	4.96 ± 0.06^ab^
CDPDP (40 mg/kg)	55.9 ± 0.43^b^	24.6 ± 0.5^b^	1.63 ± 0.10^b^	0.37 ± 0.03^ab^	5.10 ± 0.32^a^

Note: ALT, Alanine Aminotransferase/transaminase, AST, aspartate transaminase; ALP, alkaline phosphatase; BUN, blood urea nitrogen; GFR, Glomerular filteration rate. Data values represent Mean ± SD (*n* = 5). Means with different superscript (a-d) letters in the column are significantly (*p < 0.05*) different from one another.

### 3.10 Effect on biochemical levels

**FIGURE 5:**
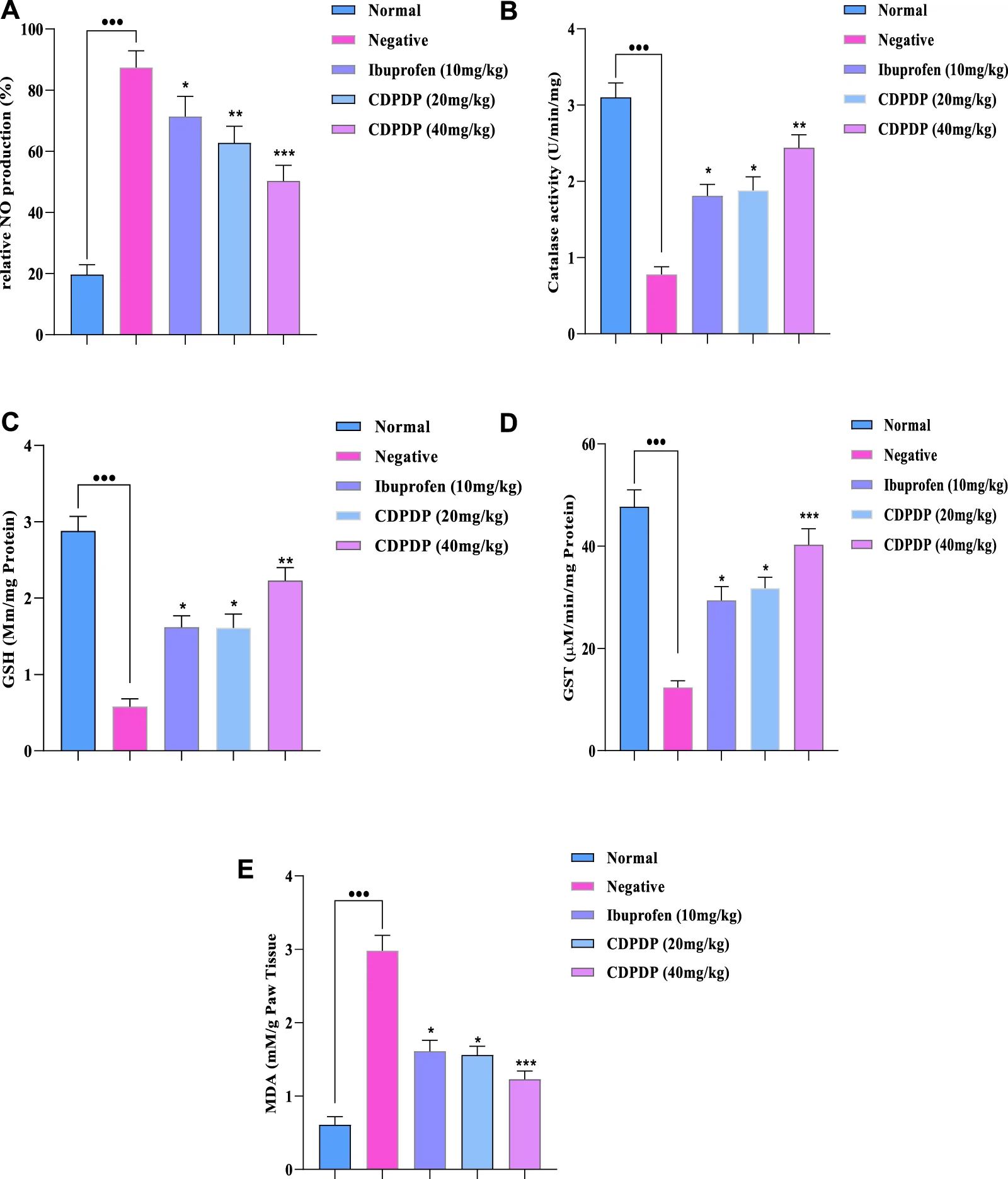
The pharmacological effect of CDPDP on the levels of nitrites and antioxidants in mice paw tissues is depicted in this graphic. The stressed compound CDPDP improved the antioxidant enzymes such as **(B)** catalase, **(C)** GSH, and **(D)** GST, while CDPDP treated RA mice model markedly attenuated the oxidative stress marker such as **(A)** NO and **(E)** MDA in comparison to other groups. NO: Nitric oxide, GSH: Glutathione, GST: Glutathione S transferase, MDA: Malonaldehyde. The data sets are expressed as the mean ± SD at *p* < 0.05 and *p* < 0.01.

Many biochemical assays, such as the release of pro-inflammatory chemicals by NO, were examined. Pro-inflammatory chemicals are enhanced during inflammation, according to several studies. Chronic autoimmune disease pathophysiology is mostly influenced by an excess of NO, which can lead to tissue destruction and some other inflammatory responses. As a result, the NO assay was initiated to determine the impact of the stress chemical (CDPDP) on NO generation. Figure 5A shows that when the stress chemical (CDPDP) was used in comparison to the control groups, NO generation was significantly reduced (*p* < 0.001). Additionally, the assays for antioxidant enzymes and oxidative stress markers were carried out to evaluate the potential of the CDPDP-treated group, which significantly increased the catalase (Figure 5B), GSH (Figure 5C), GST (Figure 5D), and MDA levels, which is an end product of lipid peroxidation, and decreased the MDA level (Figure 5E). As a result, when comparing the CDPDP (40 mg/kg) treatment to the other control groups, it was discovered to be the most effective.

#### 3.10.1 Pro-inflammatory cytokines assay

**FIGURE 6:**
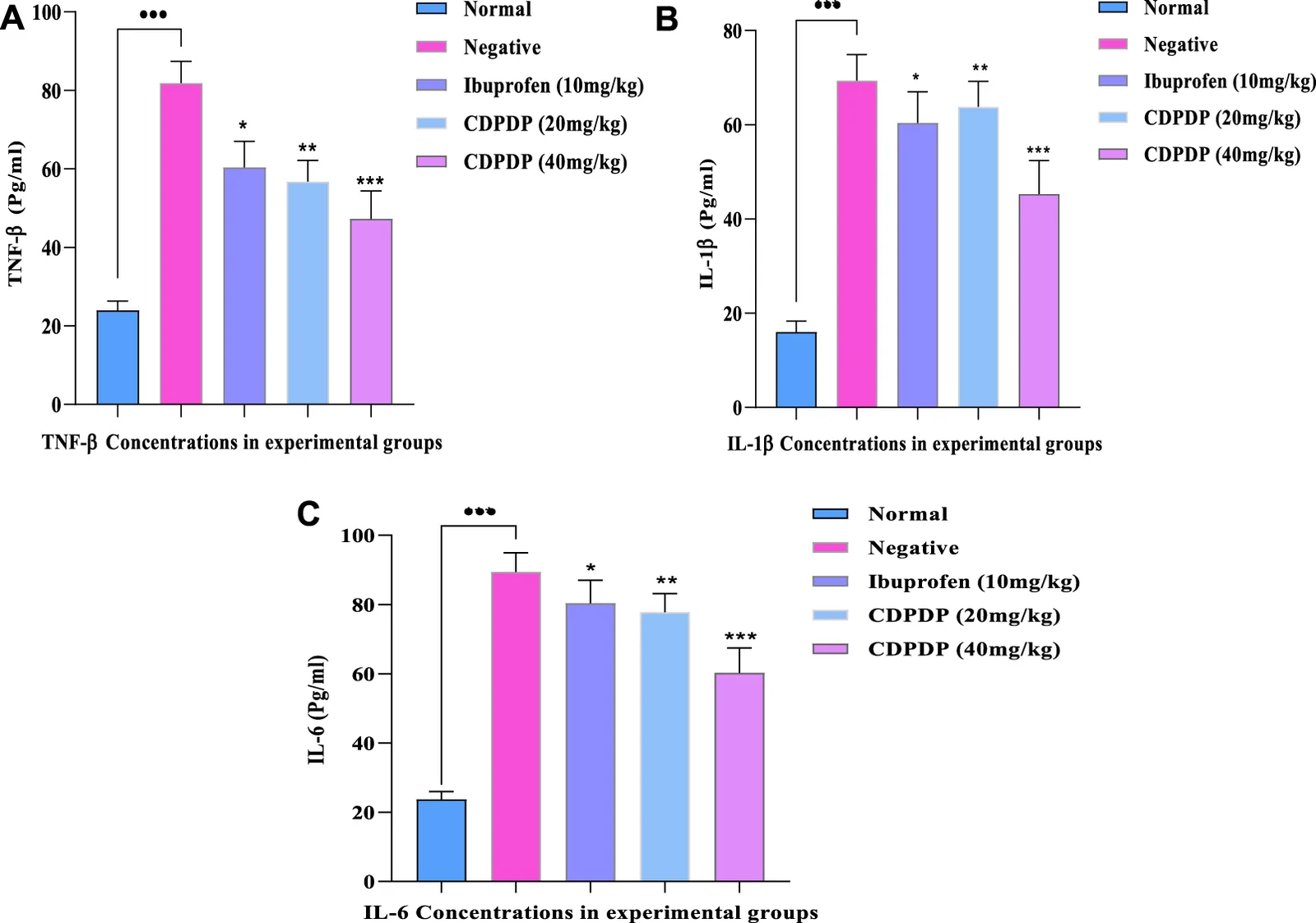
**(A)** TNF-α concentration in experimental groups **(B)** IL-Iβ concentration in experimental groups **(C)** IL-6 concentration in experimental groups. The data is expressed as the mean ± SD at *p* < 0.05 and *p* < 0.01.

Expression of TNF-α, IL-1β, and IL-6 in RA-affected tissues was significantly increased (*p* 0.001) by the CFA, which is a critical component of the amplification and progression of the disease. The effect of CDPDP on cytokine mediators such as TNF-α (Cat No: 560478), IL-1β (CAT Νο:557966), and IL-6 (CAT Νο:550950) in the paw tissue was investigated using ELISA assay kits. The findings of the assay showed the reduced expression of IL-6, IL-Iβ, and TNF-α levels in the CDPDP (40 mg/kg) group as compared to the negative and positive treated groups. CDPDP (40 mg/kg) was found to have better efficacy than the marketed drug, possibly due to its greater penetration and to its ability to be released from its target inflammatory site more quickly than from other locations (Figure 6).

### 3.11 Effect of CDPDP on liver and kidney

The results showed that CDPDP (20 and 40 mg/kg) was capable of restoring the stress-induced amended enzyme levels and biochemical parameters in the liver and kidney, such as ALT, AST, ALP, Urea, Creatinine, Albumin, and Bilirubin in arthritic mice, and that it was also effective in reducing the stress-induced altered enzyme thresholds and biochemical parameters in the liver and kidney. Management of the CDPDP pointedly improved the biochemical as well as the enzymatic outline and detailed outcomes are accessible in Table 5.

### 3.12 Cell (chondrocytes) survival assessment

To assess the safer nature of isolated compounds CDPDP against human chondrocytes cell line (CHON-001), the MTT method was followed. Chondrocytes (CHON-001) were preserved with several levels of CDPDP for 24 h, 48 h, and 72 h interval. Significant cell viability was observed on 24 h, 48 h, and 72 h exposure with CDPDP. CHON-001 cells showed significant time-dependent survival (>80%) when treated with CDPDP multiple concentrations (µg/ml). All results are displayed in Supplementary Figure S2.

#### 3.12.1 Cell viability and apoptosis analysis of chondrocytes

**FIGURE 7:**
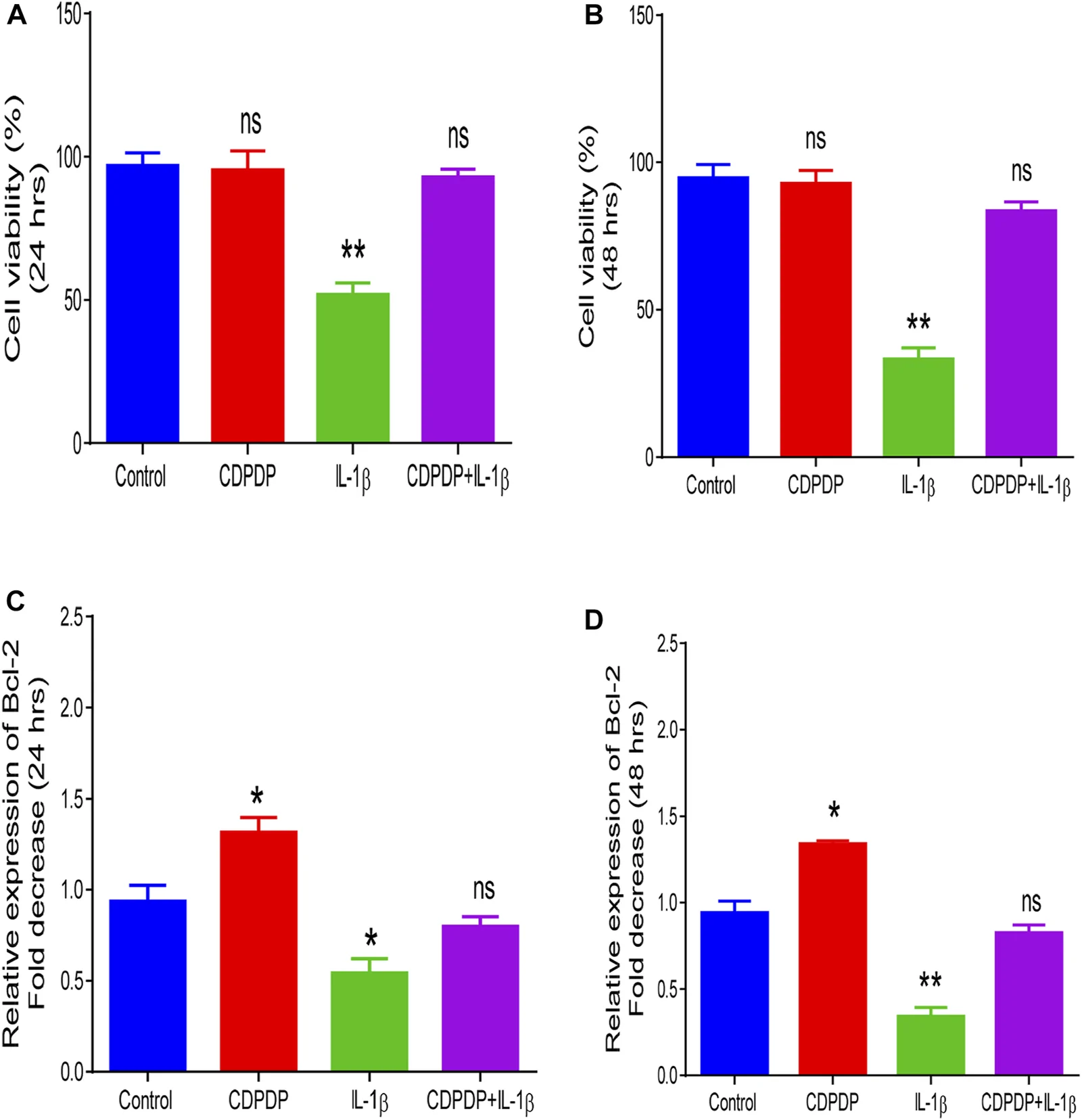
Chondrocytes were stained with trypan blue dye and trypan blue exclusion method was used to analyze CHON-001 cell viability at 24 h **(A)** and 48 h **(B)** Quantitative PCR was performed to measure **(C)** Relative Bcl-2 expression at 24 h and at **(D)** 48 h. Data is shown as mean ± SD (*n* = 3). Means with different superscripts in the columns are significantly (*p* < 0.05) different from one another.

During arthritic disease, there is a loss of cell survival signals, and enhanced apoptosis in chondrocytes, which according to research is the major factor causing arthritis (Goggs et al., 2003). IL-1β leads to activation of the inflammatory cascade in arthritis so chondrocytes treated with IL-1β are used as an *in-vitro* disease model (Chen et al., 2021). CDPDP did not negatively affect normal chondrocyte survival while IL-1β (10 ng/ml) significantly reduced the cell viability of CHON-001 cells as equated with the control (Figures 7A,B). Incubation of CHON-001 cells with CDPDP before IL-1β treatment led to an improvement in cell viability, and the non-significant difference was observed when compared with control at both time points (Figures 7A,B) Next, we analyzed relative Bcl-2 expression to get an idea of how does CDPDP affect cellular apoptosis in human chondrocyte cell lines. CDPDP-treated CHON-001 cells significantly upregulated relative Bcl-2 expression at both time points while IL-1β led to a decline in the anti-apoptosis marker. CDPDP reversed IL-1β induced decreased Bcl-2 expression, and a non-significant difference was observed between normal chondrocytes and IL-1β+CDPDP treated cells. There was a similar expression pattern at 24 h and 48 h time points (Figures 7C,D). Our results in Figure 7 signify that CDPDP can increase cell survival and inhibit apoptosis in chondrocytes during arthritic disease.

#### 3.12.2 Cartilage turnover of chondrocytes

**FIGURE 8:**
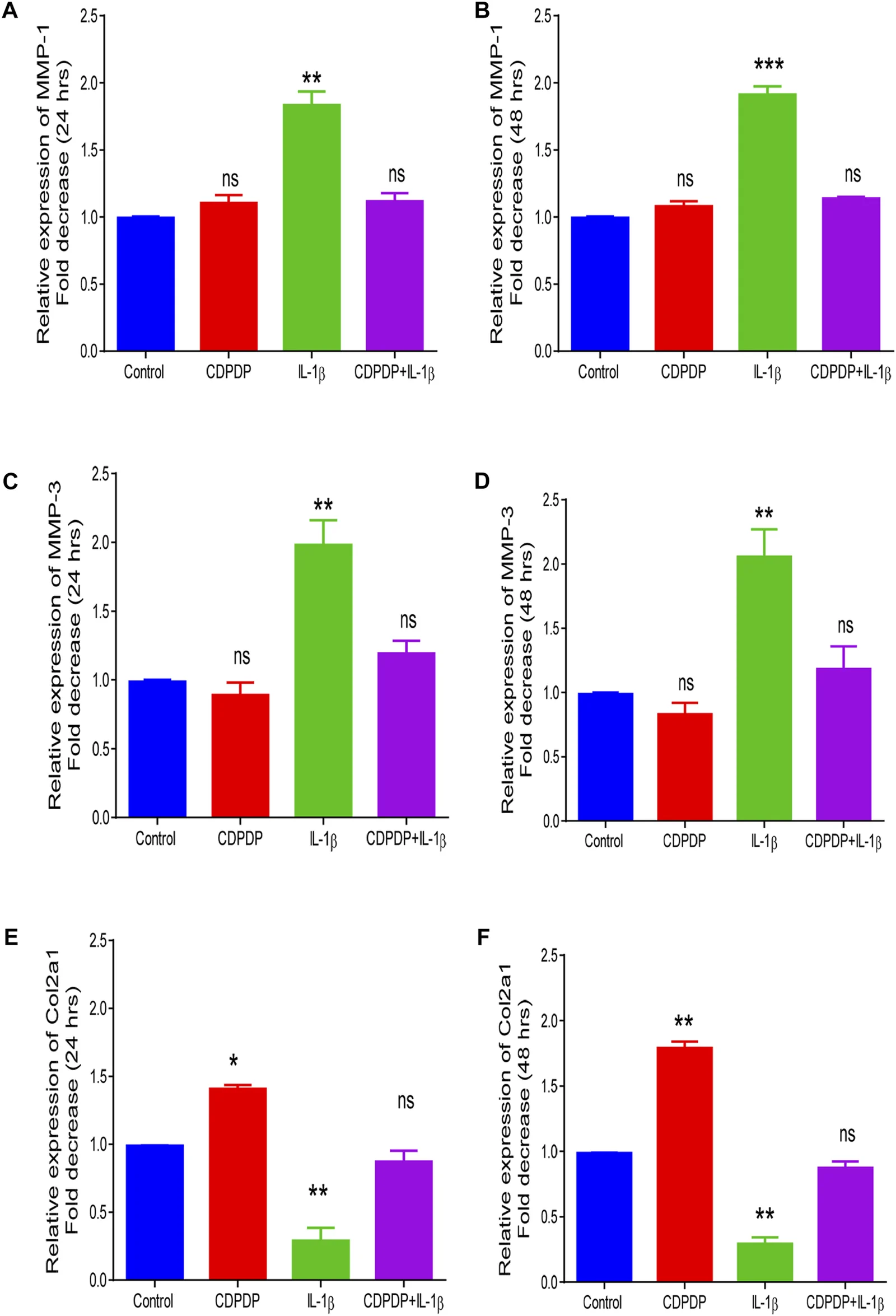
Quantitative PCR analysis chromatogram. **(A)** MMP-1 after 24 h **(B)** MMP-1 after 48 h **(C)** MMP-3 after 24 h **(D)** MMP-3 after 48 h **(E)** Col2a1 after 24 h **(F)** Col2a1 after 24 h. Data is shown as mean ± SD (*n* = 3). Means with different superscripts in the columns are significantly (*p* < 0.05) different from one another.

In arthritis, chondrocytes are responsible for the release of various matrix-degrading enzymes. IL-6 is mainly responsible for the release of these enzymes (Tseng et al., 2020). CDPDP did not alter gene expression profiles of MMP-1 and MMP3 when compared with control at 24 hs and 48 hs. On the other hand, IL-1β led to a rise in the level of these enzymes as evident from our qPCR data. Gene expression analysis of MMP-1 and MMP3 in chondrocytes incubated with CDPDP and IL-1β for 24 and 48 h also revealed no significant difference when compared with control suggesting the fact that CDPDP inhibits IL-1β induced rise in matrix-degrading enzymes concentrations. Col2a1 gene analysis revealed downregulation when treated with IL-1β but treatment with CDPDP + IL-1β significantly restored the downregulation due to IL-1β, and a non-significant difference between the control group and CDPDP + IL-1β group was observed. Our results in Figure 8 demonstrated that CDPDP can downregulate the genes involved in cartilage destruction and at the same time can upregulate protective genes like Col2a1.

#### 3.12.3 Mitochondrial biogenesis of chondrocytes

**FIGURE 9:**
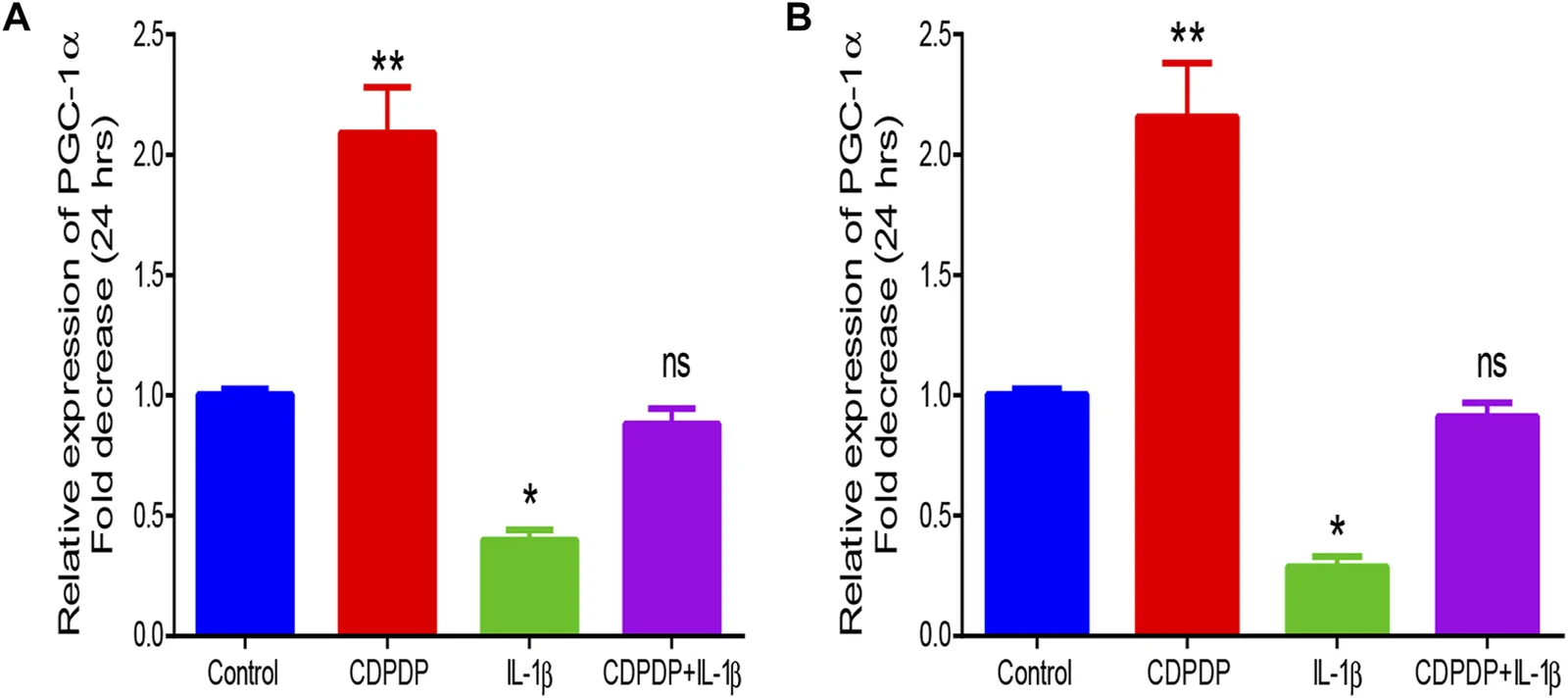
Quantitative PCR analysis chromatogram of **(A)** PGC-1α after 24 h **(B)** PGC-1α after 48 h. The data is expressed as the mean ± SD (*n* = 3) at *p* < 0.05 and *p* < 0.01.

PGC-1α gene is also called the master regulator of mitochondrial biogenesis. CDPDP upregulated the expression of PGC-1α when compared with control, while the opposite was observed when CHON-001 cells were preserved with IL-1β. CDPDP when used along IL-1β, it restored IL-1β-induced downregulation of PGC-1α Figure 9.

### 3.13 Comet assay

**FIGURE 10:**
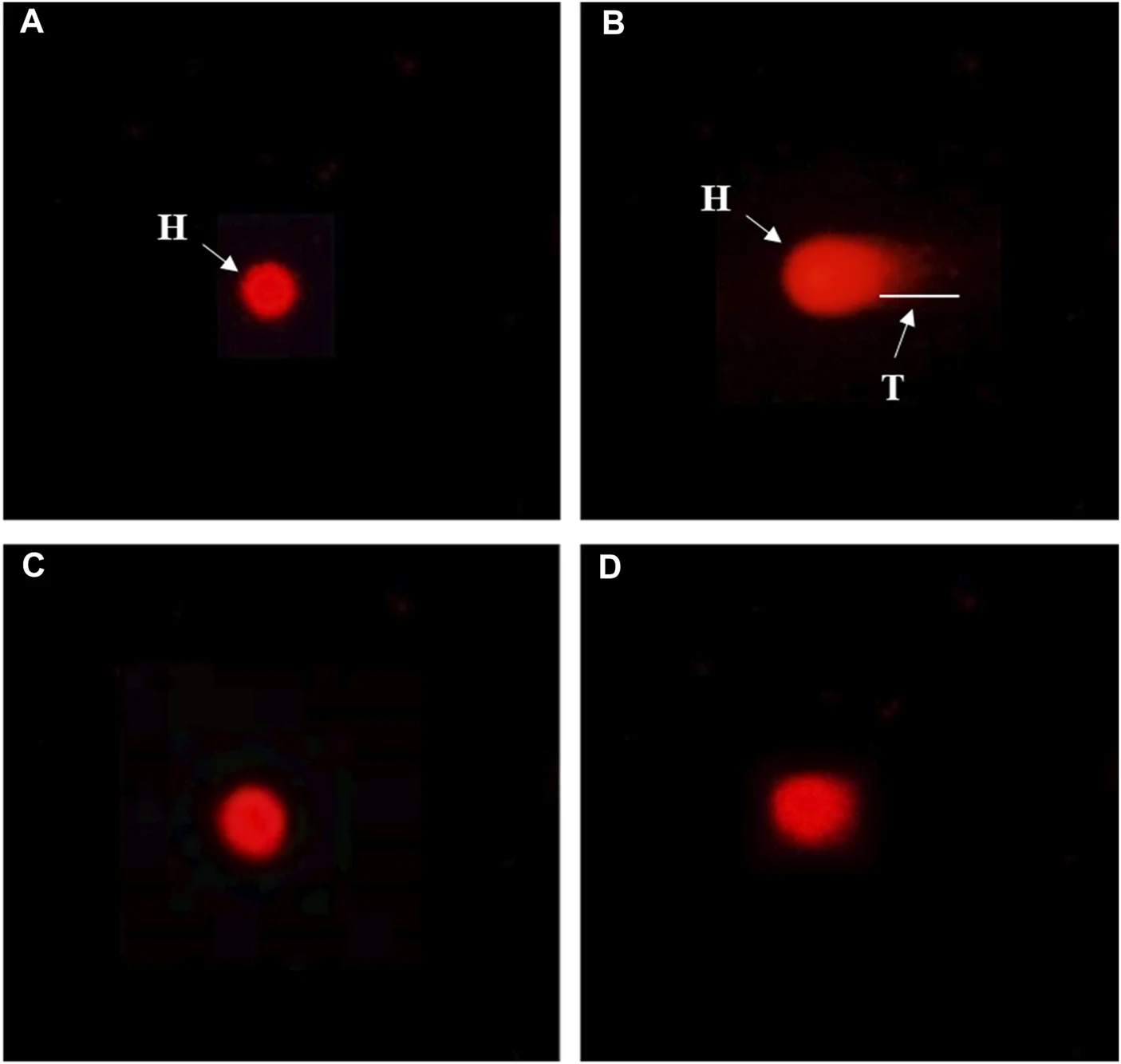
Genotoxicity evaluation of CDPDP on blood lymphocytes. Note: (**A**) The vehicle control (1% DMSO) (**B**) Ethyl methane sulfonate (20 μg/ml) (**C**) CDPDP (10 μg/ml) (**D**) CDPDP (20 μg/ml).

A comet test was performed on blood cells preserved by 10 and 20 μg/ml CDPDP sample, 20 μg/ml EMS (positive), and 1% DMSO in PBS (negative control). A CASP 1.2.3.b image processing of the photomicrographs from the fluorescence microscope was used to determine the degree of DNA damage. Tests were done on 50–100 lymphocytes per sample, measuring their length, head size, tail size, and moment of rotation. The amount of DNA found in the head and tail of lymphocytes was also evaluated. It was found that CDPDP (10 and 20 μg/ml) had no momentous effect on nuclear material, which is utilized as the standard control for genotoxicity studies. The tail length (15.0 ± 3.4 µm) of EMS-treated DNA is visibly representing the construction of nicks in nuclear content exhibiting the higher range of genotoxicity. Although no substantial toxicity to DNA was shown by any of the extracts, those with the lowest tail content were compared to the control. Figure 10; Table 6 show all of the florescent photomicrographs of the treated cells and their comet properties, respectively.

**TABLE 6:** Genotoxicity of CDPDP on blood lymphocytes was assessed using comet measurements.

Sample	Comet length (µm)	Head length (µm)	Tail length (µm)	% DNA in head	% DNA in tail	Tail moment (µm)
Control	42.6 ± 3.1	36.7 ± 2.5	5.9 ± 0.3	86.1 ± 2.8	13.9 ± 1.8^β^	0.11 ± 0.03^β^
EMS (20 μg/ml)	46.0 ± 3.6	31.0 ± 2.7	15.0 ± 3.4	67.39 ± 8.5	32.61 ± 5.8^¥^	1.37 ± 0.11^¥^
CDPDP (10 μg/ml)	40.4 ± 4.2	35.9 ± 1.8	4.5 ± 0.5	88.8 ± 2.1	11.2 ± 1.3^β^	0.11 ± 0.04 ^β^
CDPDP (20 μg/ml)	40.1 ± 2.7	32.5 ± 1.4	7.6 ± 1.0	81.1 ± 2.4	18.9 ± 1.3^¥β^	0.36 ± 0.04^¥β^

Values are expressed as Mean ± SD (*n* = 3). Means with symbol “β” indicate non-significant difference from control; “¥” from EMS, treated group according to Kruskal–Wallis test at *p < 0.05*.

### 3.14 Pharmacokinetic and toxicological properties

Prediction studies for the stress compound CDPDP’s absorption, distribution, metabolism, excretion, and toxicity (ADMET) were carried out.

#### 3.14.1 Pharmacokinetic properties

**FIGURE 11:**
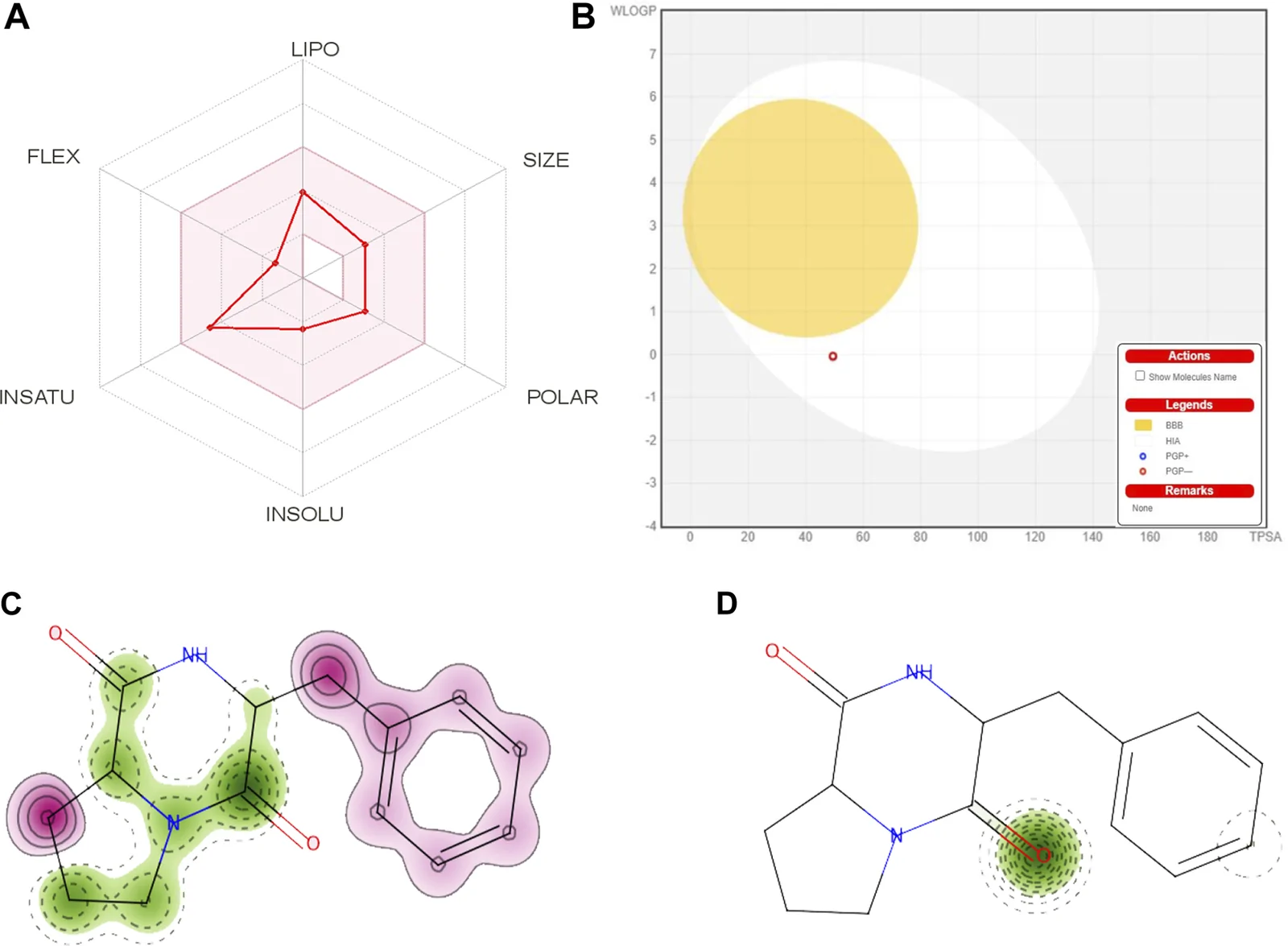
**(A)** A radar graphic showing CDPDP’s bioavailability. The pink area indicates the physicochemical region for oral bioavailability, while the red stroke shows the parameters of oral bioavailability. **(B)** Swiss ADME online web tool for CDPDP predicted BOILED-Egg plot. Map of skin sensitization of CDPDP obtained from pred-skin. **(C)** h-CLAT **(D)** LLNA. The positive and negative contributions of individual atoms or pieces to the skin sensitization have been identified. The bright pink hue shows that an atom and fragment have made a detrimental impact on skin sensitization.

The physicochemical properties of the CDPDP were looked at and separated into six major sets. Each group had a range that was good for oral bioavailability, comprising size (SIZE) 150 g/mol < MV < 500 g/mol, Insolubility (INSOLU) 0 < Log S (ESOL) < 6, Instauration (INSATU) 0.25 < Fraction Csp3 < 1, flexibility (FLEX) 0 < No. of rotatable ties <9, polarity (POLAR) 20Å2 < TPSA <130 Å^2^, and lipophilicity (LIPO) 0.7 < (Log Po/w) XLOGP3 < +5.0, respectively. As measured by the Topological Polar Surface Area (TPSA) score, the CDPDP ranged in size from 20 Å^2^ to 130 Å^2^, showing CDPDP has strong transport characteristics *in vivo* (Figure 11A) (Supplementary Table S3). For pharmaceuticals or clinical trials, one of the most important factors is that a substance has a favorable physiochemical profile, which CDPDP has shown.

In order to prevent harmful effects on the central nervous system (CNS), the blood–brain barrier must be penetrated by chemicals that operate on the CNS. Inactive molecules in the CNS should not pass the blood-brain barrier. Before entering the pharmacological or clinical trials arena, every biomolecule must have its high gastrointestinal absorption (HIA) and CNS absorption properties verified. There was reduced BBB permeability in the stress compound CDPDP, which has a high HIA value. The white part (Figure 11B) is for GI (HIA) absorption, while the yellow region (yolk) is for BBB diffusion. In the grey zone, any chemical shows that it is neither absorbed nor BBB penetrant. For example, many cancerous cells use the P-gp efflux mechanism as a drug resistance mechanism (Qu et al., 2020). CDPDP is not a P-gp substratum, hence it is not sensitive to the outflow framework of P-gp. The less skin permeation Log Kp a molecule has, the less skin permeant it is. This is especially true for the CDPDP compound, which has shown less Log Kp. In addition, five main cytochromes (CYP) isoforms may be predicted using this method. Nearly 75% of commercially available pharmaceuticals are metabolized by this enzyme isoform, which plays a critical role in excretion. When one of these isoforms is suppressed, significant drug-drug interactions occur. For example, the CDPDP did not affect the cytochrome isoform, and because it rapidly metabolizes, there are no interactions with these chosen cytochromes it might interfere with (Table 7). Dosing rates for steady-state concentrations are determined using the sum of renal and hepatic clearance. There was a problem with the CDPDP clearance value. Organic Cation Transporter 2 (OCT2) intermediates may have an influence on the unfavorable interactions that occur when OCT2 inhibitors and substrates are used together. CDPDP was found to be an OCT2 non-substrate in a recent study.

**TABLE 7:** Predicted pharmacokinetics parameters of CDPDP and Ibuprofen.

Properties	Parameters	CDPDP	Ibuprofen
Absorption	Water Solubility	−2.59	−3.36, S^a^
	GI^b^	81.53	94.01
	Log *K*_p_ (Skin permeation) cm/s	−6.80	−5.07
Distribution	BBB^a^	−0.075	0.302, Yes
	CNS permeation (Log PS)	−2.519	−1.695
	V_D_ ^c^ (human)	−0.168	−0.877
Metabolism	CYP1A2 inhibitor	No	No
	CYP2C9 inhibitor	No	No
	CYP2C19 inhibitor	No	No
	CYP3A4 inhibitor	No	No
	CYP2D6 inhibitor	No	No
Excretion	Total Clearance (log ml/min/kg)	0.282	0.263
	Renal OCT2 substrate	Yes	No

^a^ Blood-brain barrier.

^b^ Gastrointestinal.

^c^ Volume of distribution.

#### 3.14.2 Toxicity assessment

Drugs must be evaluated for their toxicological profile when they first enter the clinical trials or production stages of the pharmaceutical industry. To determine the CDPDP’s potential toxicity, it has been tested on several animals, including humans and oral mice, and in the environment) (Supplementary Table S4). This test is used to examine a compound’s capacity to cause mutations in other organisms. Ames’ hazardous classification was given to CDPDP, suggesting that they have been unlikely to be carcinogenic. Cardiovascular arrhythmias may be caused by the inhibition of potassium channels expressed by the human electroreceptor gene (hERG). According to the findings, CDPDP inhibits the expression, including both hERG I and hERG II. Aside from that, the molecule CDPDP was expected to be non-hepatotoxic, so it will not induce drug-induced liver damage. Besides considering environmental poisonousness, the online web browser GUSAR was utilized. The environmental toxicity was projected *via* an online web server using 96-h fathead minnow 50 percent fatal concentration, 48-h Daphnia Magna 50% lethal intensity, *Tetrahymena* pyriformis 50% inhibition zones concentration, and bio-concentration parameters, among other variables. With GUSAR’s environmental toxicity prediction, CDPDP is within the application area of models in every instance. It is clear from the toxicity outline that the stress compound CDPDP has a strong protection outline, particularly in terms of hepatotoxicity.

#### 3.14.3 Cardiac toxicity

The blocking of the hERG K+ channels is associated with deadly cardiac arrhythmias (Braga et al., 2015), and the FDA requires that every biomolecule that is considered as a therapeutic candidate be tested for hERG safety before being approved (Supplementary Figure S3). shows the prediction of CDPDP that was created after cardiac toxicity was predicted by pred-hERG and is based on this prediction. The positive and negative contributions of individual atoms or pieces to the hERG blockade have been identified. The bright pink hue shows that an atom and fragment have made a detrimental impact on the hERG blockade. The pred-hERG predictions projected that the stress compound CDPDP would not be cardiotoxic, with a 50 percent confidence level in this prediction. The findings have shown that CDPDP is less dangerous in terms of cardiac toxicity.

#### 3.14.4 Skin sensitization

Chemo-induced skin sensitization is a complicated immunological condition that has a significant impact on both the sufferer’s well-being and their capacity to function. Despite considerable advances in the development of innovative methods of evaluating the skin sensitization capability of chemical compounds, there seems to be no *in vitro* assay that corresponds well with human data. Computational QSAR models can be used to quickly screen chemicals for toxicity and provide valuable information. The stressed compound CDPDP was evaluated for its possible skin sensitization in the *in-vitro* human cell line activation test (h-CLAT) OECD442E and *in-vivo* local lymph node assay (LLNA) OECD429. The cellular response of CDPDP was evaluated against dendritic cells for the induction of inflammatory cytokines and the mobilization of dendritic cells. The h-CLAT- OECD442E results have shown that CDPDP was conducted as a non-sensitizer with a confidence score of 65.1% (Figure 11C). Furthermore, *in-vivo* (LLNA) evaluation of CDPDP was evaluated for tissue/organ response against the proliferation of antigen-specific T-cells (OECD429). The results have shown that CDPDP was conducted as a non-sensitizer with a confidence score of 98.5% (Figure 11D). From both cellular response and tissue/organ response, it also proved that the stressed compound CDPDP is a non-sensitizer for the histocompatibility complex represented by dendritic cells.

### 3.15 Docking analysis

**FIGURE 12:**
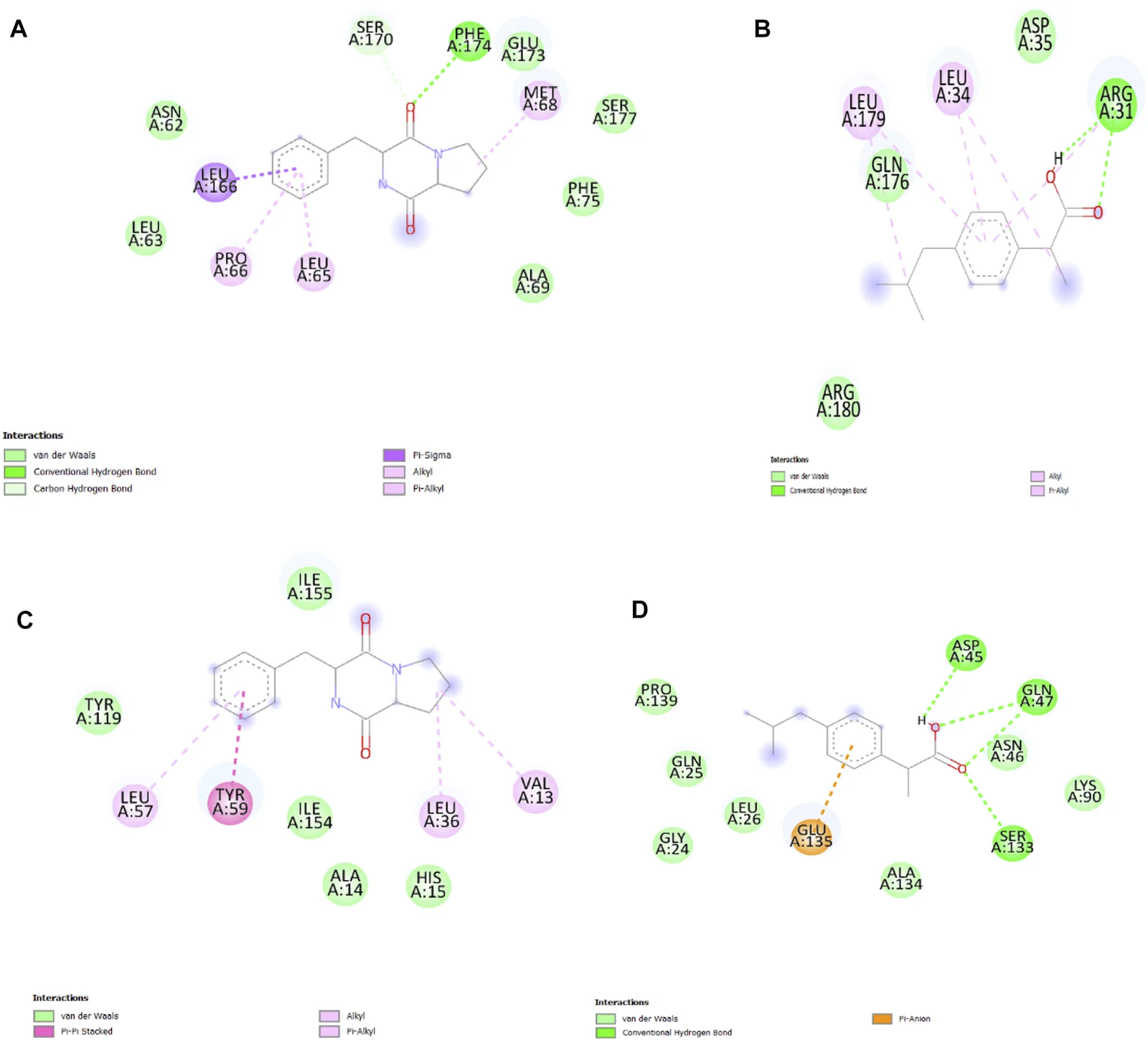
**(A)** 2D interaction of CDPDP with Interleukin-6, **(B)** 2D interaction of ibuprofen with Interleukin-6. **(C)** 2D interaction of CDPDP with TNF-α **(D)** 2D interaction of ibuprofen with TNF-α.

The key residues of the selected active binding pocket of Interleukin-6 protein (PDB ID: 1il6) and Tumor Necrosis Factor-alpha (PDB ID: 1tnf) against CDPDP were found. Main ligand-protein interactions of CDPDP against Interleukin-6 protein include LEU166, PRO66 and MET68 in pi-stacking interactions, and PHE174 in hydrogen-bonding interaction (Figure 12A). In the case of Tumor Necrosis Factor-alpha TYR59, pi-stacking interactions were found (Figure 12C). To validate the docking protocol used in this study, the reference ligand, ibuprofen, was initially docked into the binding pocket of the Interleukin-6 protein (PDB ID: 1il6) and Tumor Necrosis Factor-alpha (PDB ID: 1tnf) protein domain. The result demonstrated a suitable binding pose of the reference ligand with a docking score of −17.2 kcal/mol. The interaction between ibuprofen and the binding sites of Interleukin-6 protein mainly involved the amino acids LEU179, and LEU34 in pi-stacking interactions, and ARG31 in hydrogen-bonding interactions (Figure 12B). The interaction between ibuprofen and the binding sites of Tumor Necrosis Factor-alpha protein mainly involved the amino acids ASP45, GLN47, AND SER133 in hydrogen-bonding interaction (Figure 12D).

### 3.16 Molecular dynamics and simulation

MDS was performed for the CDPDP- Interleukin-6, CDPDP- Tumor Necrosis Factor-alpha, Ibuprofen- Interleukin-6, and Ibuprofen-Tumor Necrosis Factor-alpha structure complexes upto100 ns The parameters explored for analyses such as RMSD, RMSF, protein-ligand contact, RoG, and binding free energy.

#### 3.16.1 RMSD analysis

**FIGURE 13:**
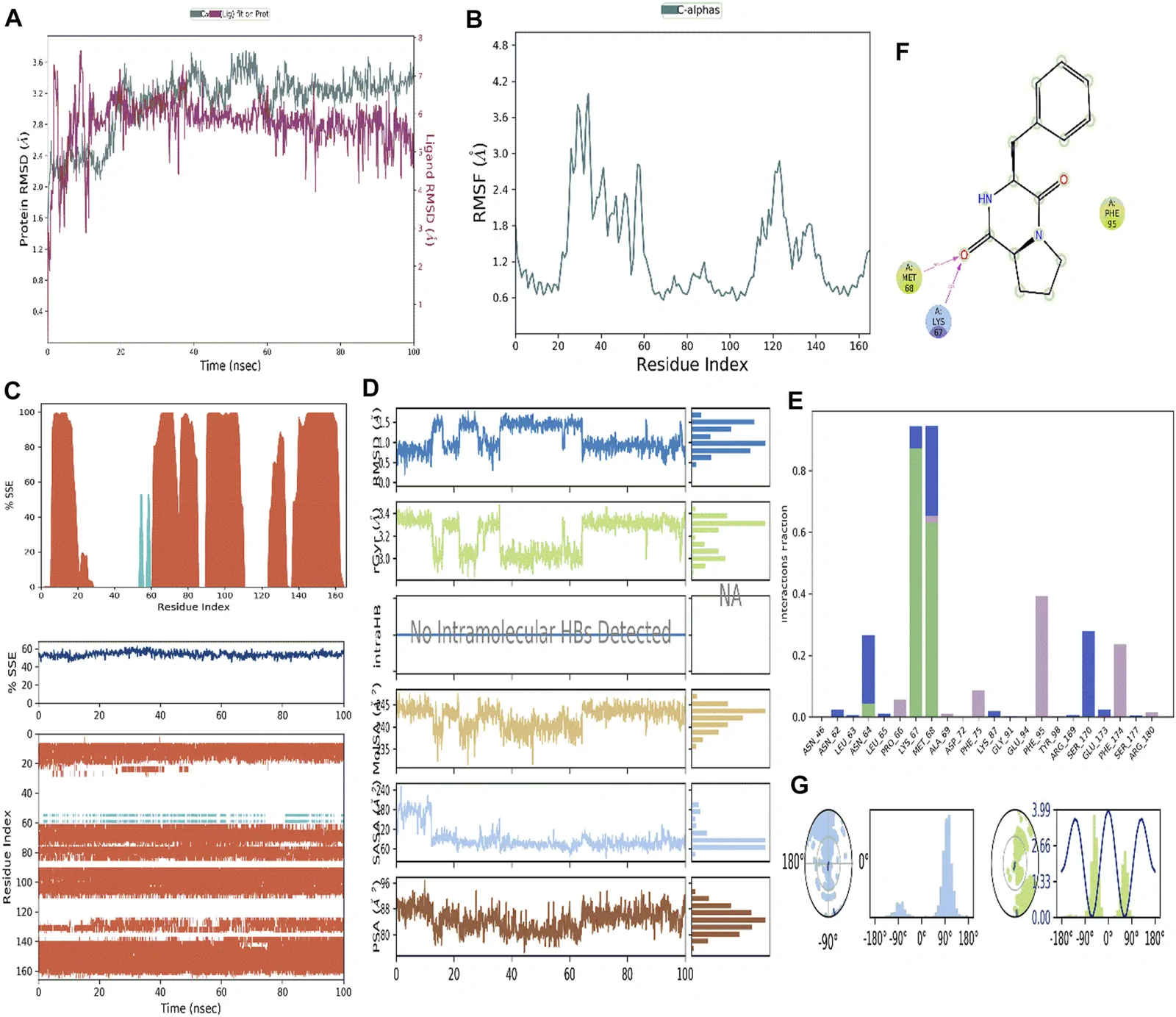
**(A)** RMSD analysis of CDPDP-IL-6 protein complex **(B)** Root Mean Square Fluctuation (RMSF) RMSF analysis of CDPDP-IL-6 protein complex **(C)** Protein Secondary Structure element distribution by residue index throughout the protein structures complexed with ligand CDPDP- IL-6 **(D)** Ligand properties with IL-6 protein **(E)** Protein-ligand contact histogram of CDPDP-IL-6 **(F)** Protein-ligand interaction of CDPDP-IL-6 **(G)** Ligand torsional plot of CDPDP.

The RMSD study was used to determine the stability of the simulation results. During the simulation, the protein’s RMSD graph (left Y-axis) can provide insight into its structural conformation, while the ligand’s RMSD graph (right Y-axis) indicates the stability of the ligand concerning a specific protein and its ligand-binding pocket (Figure 13A). CDPDP-Interleukin-6 protein-ligand complex remained stable and within acceptable RMSD limits throughout 100 ns simulation (Figure 13A).

#### 3.16.2 RMSF assay

The RMSF is a measure of the macromolecule’s particle deviation. It describes the pliability and stiffness of the protein structure. Since the N and C-terminal residues tend to fluctuate the most in MD trajectories, these residues have higher peaks (Figure 13B). Binding site residues with low RMSF values show that ligand-binding is highly stable. The percentages of helix and strand in CDPDP- IL-6 were discovered to be 52.5% and 1.28%, respectively, while the overall secondary structure elements (SSE) were found to be 53.77% (Figure 13C).

#### 3.16.3 Simulation event analysis of ligand properties

The properties of the ligand which is complexed with the protein of IL-6 during simulation studies (Figure 13D) are as follows ligand RMSD was little fluctuating between 0.6 and 1.4 with an average of 0.8 $A^{0}$ which proves that compound is stable with the protein active site. Furthermore, the structural compactness of the protein was checked by the radius of gyration (Rg). The ligand showed very good stability from 0–40 and 60–100 ns but was little fluctuated from 40 to 60 ns. There was no intra-hydrogen bond found in this complex. Molecular surface area (MoLSA) was found to be maintained in the zone of 230–245, there were little fluctuations but later the ligand showed good stability. The solvent-accessible surface area (SASA) also showed some fluctuations in the start till 40ns but after that showed good stability till 100 ns The polar surface area was also maintained in the zone of 78–84.

#### 3.16.4 Molecular interactions of protein-ligand

The interaction of the target protein with the ligand was monitored during the simulation. These interactions were categorized into four types: 1) Hydrogen Bonds, 2) Hydrophobic, 3) Ionic, and 4) Water bridges (Figures 13E,F).

According to the findings of this investigation, hydrogen bonds, water bridges, and hydrophobic interactions were the most important ligand-protein interactions observed. In CDPDP-IL-6, the complex showed hydrophobic interaction with PRO_66, PHE_95, and PHE_174 whereas LYS_67 and MET_68 were key in terms of hydrogen bonding (Figure 13E). While the hydrophobic interactions and positive charge on the structure of the ligand are shown in Figure 13F. Furthermore, the compound CDPDP torsions plot describes the no of rotatable bonds in the ligand from 0.00ns to 100 ns. Figure 13G depicts a 2 d depiction of the ligand in which rotatable bonds are color labeled according to their orientation. There were two rotatable bonds found in the structure consuming the energy at 3.99 kcal/mol.

### 3.17 MM-GBSA calculations

Total binding free energy was calculated for the CDPDP with IL-6 as shown in Supplementary Table S5. In IL-6, the binding free energy was calculated as −55.46 kcal/mol for the CDPDP.

## 4 Discussion

This research focused on isolating a metal-triggered anti-arthritic compound CDPDP from actinobacteria by abiotic stress. Furthermore, evaluating the *in vitro and in-vivo* pharmacological profile of CDPDP as an anti-inflammatory, anti-pyretic and anti-oxidant agent, including *In-silico* studies of ADMET, molecular docking and MD simulations strengthened its profile.

Metal-triggering technique is one of the new techniques which increases the chances for new secondary metabolite production. As proved by our previous findings, Metals-triggered compounds are generally absent in the normal culture method but after inducing abiotic stress (heavy metals) the new compound with its unique carbon skeletons appeared in stressed culture (Ul Hassan et al., 2021). The microorganisms sometimes show resistance towards the metals and therefore need to either increase the concentration of metals or use the combination of two or more metals to trigger the sleeping genes (Hassan et al., 2017; Shams ul Hassan et al., 2019; Ul Hassan et al., 2021). Our research shows that the actinobacteria strain initially did not respond to copper with different concentrations. However, after the addition of Nickel ions, the bacteria responded to the metal ions in its environment and elicited a new peak as CDPDP.

Nowadays, many natural product researchers have driven their research toward untapping the secondary metabolites with promising anti-inflammatory and anti-oxidant properties (Mahmood et al., 2022; Qazi et al., 2022; Razzaq et al., 2022). When an allergic reaction occurs during croton-induced ear and anal inflammation, histamine is released, which results in vasodilation, edoema, increased vascular permeability, and the recruitment of eosinophils (Li et al., 2022). In response the rat’s anal sphincter’s smooth muscles acquire inflammation. It controls the function of leukocytes as well as their movement, together with the proliferation of T cells and B cells, and secretion of lysosomal enzymes in neutrophils (Majid et al., 2018). During the process of histamine-induced edoema, endothelial nitric oxide synthase, also known as eNOS, is extremely important. Because NO is directly responsible for the macromolecular surge generated by histamine inflammation, reducing NO levels may help to reduce inflammation (Song and Wu, 2022). This is because NO is immediately accounted for by the inflammatory process (Batool et al., 2017). Oral administration of CDPDP is used in this study to generate a systemic effect, with the goals of stabilizing the membrane of mast cells and reducing the production of NO and histamine. The adjuvant-induced inflammatory rat model has been used widely as a classical animal model. This model has several of the typical aspects as in human arthritic patients, including immunological and histological findings. Primary and secondary chronic arthritis can be induced by adjuvant-induction, which activates the cell-mediated immune response and promotes immunoglobulin synthesis in organisms. As a result of the high levels of pro-inflammatory cytokines released during adjuvant induced inflammation. Osteoclast differentiation, inflammation, and bone degradation are all influenced by pro-inflammatory cytokines and interleukins (Majid et al., 2018). The production of metallo-proteinases and the proliferation of synovial cells, both of which lead to cartilage deterioration, can be stopped by blocking TNF-, IL-1, IL-6, and NO. CDPDP with substantial inhibition capacity of these cytokines has significantly reduced CFA-induced inflammatory complications.

ADMET is a crucial stage for every kind of biomolecule before its biotransformation into a drug (Hassan et al., 2022). According to the ADMET profile of CDPDP, its absorption and distribution were moderate; CDPDP is safe in cases of hepatotoxicity, cardiotoxicity, and cytochrome inhibition. Furthermore, CDPDP has revealed that it is not a P-gp (P-glycoprotein) substrate; therefore, CDPDP is not susceptible to the efflux mechanism of P-gp, and many cancer cell lines utilize that as a drug resistance mechanism. CYP enzymes play a crucial role in drug excretion, and these isoforms are metabolizing almost 75% of market-available drugs. Inhibition of any of these isoforms results in causing some significant pharmacokinetics-based drug-drug interactions (Shams ul Hassan et al., 2021) CDPDP has not inhibited any CYP enzymes, which means MVL cannot create drug-drug interactions for those CYP enzyme-targeted drugs.

As evident from our study, CDPDP has shown encouraging results in terms of IL-6 and TNF-α inhibition in the sense of molecular docking. Therefore, in these ways, special attention should be placed on investigating this process’s therapeutic importance. Further molecular docking simulations proved that CDPDP has good binding potential with IL-6 and binds within the active gorge.

Thus, the current study revealed the potential advantages of the metals-triggered compound CDPDP against arthritis and may be useful against different diseases. The research relied on *in vitro*, *in-vivo*, and computational tools that documented pharmacological properties and bioactivities predictions. Moreover, clinical studies are necessary to confirm the findings of the present work. Nonetheless, the results of this work will provide future guidance for the design and development of new lead compounds as anti-arthritic, anti-pyretic and anti-oxidant agents.

## 5 Conclusion

In the current research, the abiotic metal (Ni^2+^) stress was employed to isolate a stressed molecule from actinobacteria, a natural substance called CDPDP. CDPDP’s *in vivo* clinical profile as an anti-inflammatory medication against rheumatoid arthritis was further enhanced by ADMET investigations *in silico*, molecular docking, and simulation studies. The use of the metal-stress technique paves the way for the discovery of new secondary metabolites, as it enables researchers to focus on the most promising areas. Due to the prior discovery that stress-elicited compounds are often absent from standard cultures, this new chemical with its characteristic carbon skeletons appeared in the stressed culture method. As a result, they must either increase the concentration of metals or combine two or more different metals to activate the genes that have been dormant for a long time. Secondary metabolites with potential anti-inflammatory activities have become the focus of many natural product researchers in the last several years. As mentioned in Tables 1, 2 the CDPDP in its (40 mg/kg) dose has shown promising anti-inflammatory activity against R.A as compared to positive control Ibuprofen. A strong anti-inflammatory profile is demonstrated by these findings for CDPDP. Furthermore, for the biochemical assays and enzymatic studies as mentioned in Figures 7, 8. The compound CDPDP has shown promising results against TNFα, IL-6, GSH assay, GST assay, catalase, and NO production. In addition, the histopathological studies and X-ray reports have proved that CDPDP (40 mg/kg) remarkably finished the R.A and increased the fluid between joints. The compound CDPDP also showed a safe profile for the kidney and liver after the dosage of the compound to mice evaluated after 6 h of administration. According to our findings, CDPDP has demonstrated promising outcomes in the context of molecular docking for the suppression of IL-6. As a result of these considerations, this process’s therapeutic significance must be examined.

## Data Availability

The original contributions presented in the study are included in the article/Supplementary Material, further inquiries can be directed to the corresponding authors.
