# Correction: Metals-triggered compound CDPDP exhibits anti-arthritic behavior by downregulating the inflammatory cytokines, and modulating the oxidative storm in mice models with extensive ADMET, docking and simulation studies

**DOI:** 10.3389/fphar.2026.1958910

**Published:** 2026-10-01

**Authors:** Syed Shams ul Hassan, Syed Qamar Abbas, Ishaq Muhammad, Jia-Jia Wu, Shi-Kai Yan, Fawad Ali, Muhammad Majid, Hui-Zi Jin, Simona Bungau

**Affiliations:** 1 Shanghai Key Laboratory for Molecular Engineering of Chiral Drugs, School of Pharmacy, Shanghai Jiao Tong University, Shanghai, China, Shanghai, China; 2 Department of Natural Product Chemistry, School of Pharmacy, Shanghai Jiao Tong University, Shanghai, China; 3 Department of Pharmacy, Sarhad University of Science and Technology, Peshawar, Pakistan; 4 Department of Pharmacy, Kohat University of Science and Technology, Kohat, Pakistan; 5 Faculty of Pharmacy, Hamdard University, Islamabad, Pakistan; 6 Department of Pharmacy, Faculty of Medicine and Pharmacy, University of Oradea, Oradea, Romania

**Keywords:** actinobacteria, inflammation, arthritis, anti-oxidant, admet, molecular docking

In the published article, the reference cited in section 3.14.3 “Cardiac toxicity” in support of the statement “The blocking of the hERG K+ channels is associated with deadly cardiac arrhythmias” was erroneously written as “Xue, F., Cheng, J., Liu, Y., Cheng, C., Zhang, M., Sui, W., et al. (2022). Cardiomyocyte-specific knockout of ADAM17 ameliorates left ventricular remodeling and function in diabetic cardiomyopathy of mice. Signal Transduct. Target. Ther. 7, 259. doi:10.1038/s41392-022-01054-3”.

It should be “**Braga et al., 2015**” which is already available in the reference list.

In the published article, in section 3.12.2 “Cartilage turnover of chondrocytes,” the sentence was erroneously given as: “IL-6 is mainly responsible for the release of these enzymes (Tseng et al., 2020; Xu et al., 2021).” The citation “**Xu et al., 2021**” was erroneously included and does not support this statement.

This has been corrected to read:

“IL-6 is mainly responsible for the release of these enzymes (**Tseng et al., 2020**).”

The corresponding reference, “Xu, S., Tao, H., Cao, W., Cao, L., Lin, Y., Zhao, S.-M., et al. (2021). Ketogenic diets inhibit mitochondrial biogenesis and induce cardiac fibrosis. Signal Transduct. Target. Ther. 6, 54. doi:10.1038/s41392-020-00411-4,” has been removed from the text and reference list.

The original version of this article has been updated.

