# Supplementary material for: Metals-triggered compound CDPDP exhibits anti-arthritic behavior by downregulating the inflammatory cytokines, and modulating the oxidative storm in mice models with extensive ADMET, docking and simulation studies
Source: Front Pharmacol. 2022 Nov 23;13:1053744. doi: 10.3389/fphar.2022.1053744 (PMC9727203; doi:10.3389/fphar.2022.1053744)
Supplement: Supplementary file 1 [file Table1.docx]

**Supplementary Data**

**
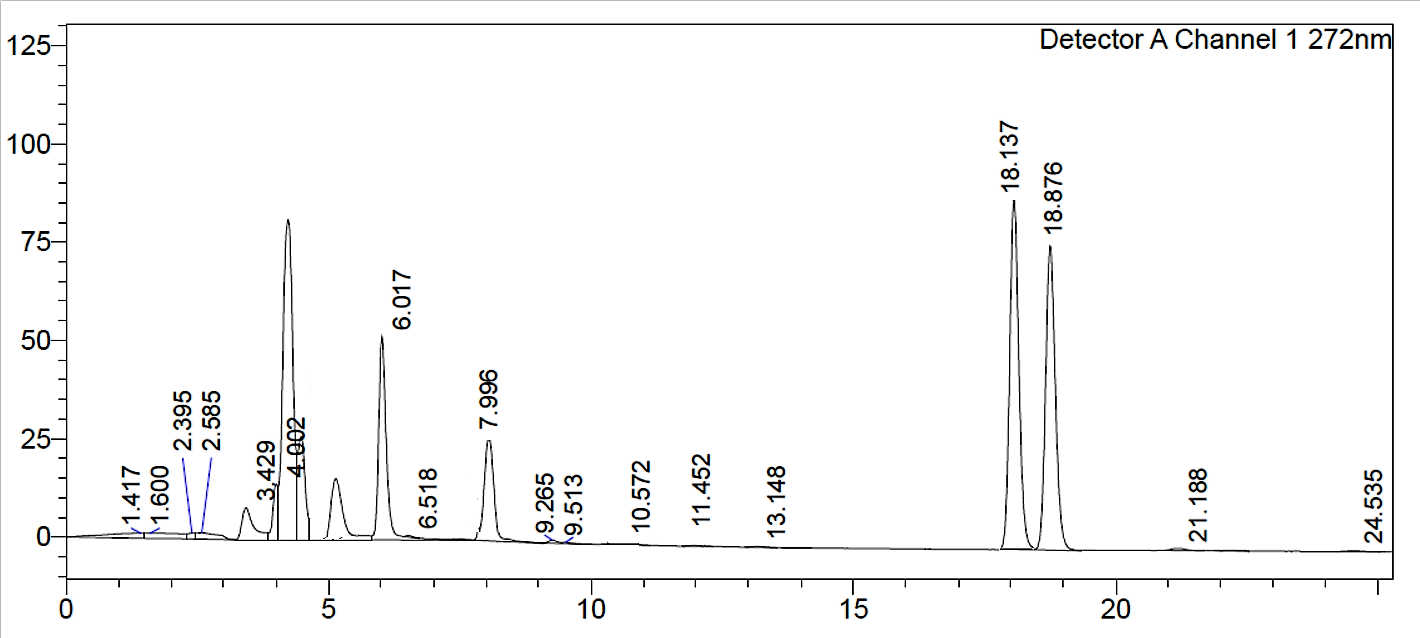
**

**Figure. S1.** HPLC chromatogram of metal triggered compound

**Figure. S2.**  Effect of CDPDP on the viability of human chondrocytes cell line (CHON-001)

**Note:** At 24, 48, and 72 hours after exposure to CAPDP, MTT assays were used to gauge the survivability of CHON-001 cells. Data is presented as mean±SEM of % cell viability (n = 3) at p < 0.05.


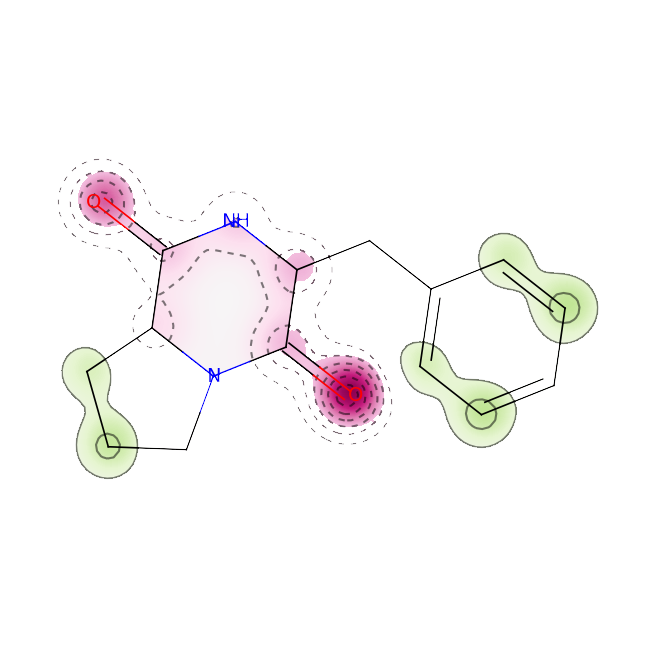


**Figure S3** Using pred-hERG, showing CDPDP's cardiac toxicity map.

**Table S1.** primer sequence of qPCR.

| Primer name |  | Sequence (5'-3') |
| --- | --- | --- |
| *In vitro* (Homo Sapiens) | | |
| *Bcl-2* | F | GGTGGGGTCATGTGTGTGG |
|  | R | CGGTTCAGGTACTCAGTCATCC |
| *PGC-1α* | F | TCTGAGTCTGTATGGAGTGACAT |
|  | R | CCAAGTCGTTCACATCTAGTTCA |
| *MMP1* | F | GGGGCTTTGATGTACCCTAGC |
|  | R | TGTCACACGCTTTTGGGGTTT |
| *MMP3* | F | CTGGACTCCGACACTCTGGA |
|  | R | CAGGAAAGGTTCTGAAGTGACC |
| *COL2A1* | F | TGGACGCCATGAAGGTTTTCT |
|  | R | TGGGAGCCAGATTGTCATCTC |

**Table S2.** Cycling temperature of qPCR.

Vol:20µl

| Hold stage | PCR stage | | | Melt curve stage | |  |
| --- | --- | --- | --- | --- | --- | --- |
| 95°C  05:00 | 95°C  05:00 | 60°C  00:30 | 72°C  00:30 | 95°C  05:00 | 60°C  01:00 | 95°C  00:15  camera |
| Step 1 | Step 1 | Step 2 | Step 3 | Step 1 | Step 2 | Step 3 |

**Table S3.** The physicochemical limitations and lipophilicity possessions of CDPDP and Ibuprofen.

| **Belongings** | **Parameters** | **CDPDP** | **Ibuprofen** |
| --- | --- | --- | --- |
| Physicochemical properties | MW^a^ (g/mol) | 244.29 | 206.28 |
|  | Rotatable bonds | 0 | 4 |
|  | HBA^b^ | 2 | 2 |
|  | HBD^c^ | 1 | 1 |
|  | Fraction Csp3 | 0.43 | 0.46 |
|  | TPSA^d^ | 49.41 | 37.30 |
| Lipophilicity  Log *P_o/w_* | iLOGP | 2.03 | 2.26 |
|  | XLOGP3 | 1.39 | 3.50 |
|  | MLOGP | 1.18 | 3.13 |
|  | Consensus | 1.23 | 3.04 |

**Table S4.** Predicted toxicity profile of CDPDP and Ibuprofen.

| **Parameters** | **CDPDP** | **Ibuprofen** |
| --- | --- | --- |
| Ames toxicity | No | No |
| Max. tolerated dose (human) (log mg/kg/day) | -0.116 | 1.063 |
| hERG I inhibitor | No | No |
| hERG II inhibitor | No | No |
| Hepatotoxicity | No | Yes |
| Skin Sensitisation | No | Yes |
| Bioaccumulation factor Log10 (BCF) | 0.375 | 1.207 |
| *Daphnia magna* LC50 -Log10 (mol/L) | 4.905 | 4.510 |
| Fathead Minnow LC50 Log10 (mmol/L) | -0.485 | -1.373 |
| *Tetrahymena pyriformis* IGC50 -Log10 (mol/L) | 0.159 | 0.873 |

**Table S5.** MM-GBSA for protein Interleukin-6

| **Ligand** | **Total** | **Coulomb** | **Covalent** | **H-bond** | **Lipo** | **Packing** | **SolvGB** | **VDW** |
| --- | --- | --- | --- | --- | --- | --- | --- | --- |
| CDPDP | -55.46266289 | -8.018633442 | 0.374379367 | -0.214999607 | -24.66105641 | -0.002466531 | 14.07663287 | -37.01651913 |
